# Influence of Long Non-Coding RNA in the Regulation of Cancer Stem Cell Signaling Pathways

**DOI:** 10.3390/cells11213492

**Published:** 2022-11-04

**Authors:** Kevina Sonawala, Satish Ramalingam, Iyappan Sellamuthu

**Affiliations:** Department of Genetic Engineering, SRM Institute of Science and Technology, Kattankulathur, Chennai 603202, India

**Keywords:** non-coding RNAs, lncRNAs, cancer stem cells, signaling pathways, CSC signaling pathways

## Abstract

Over the past two decades, cancer stem cells (CSCs) have emerged as an immensely studied and experimental topic, however a wide range of questions concerning the topic still remain unanswered; in particular, the mechanisms underlying the regulation of tumor stem cells and their characteristics. Understanding the cancer stem-cell signaling pathways may pave the way towards a better comprehension of these mechanisms. Signaling pathways such as WNT, STAT, Hedgehog, NOTCH, PI3K/AKT/mTOR, TGF-β, and NF-κB are responsible not only for modulating various features of CSCs but also their microenvironments. Recently, the prominent roles of various non-coding RNAs such as small non-coding RNAs (sncRNAs) and long non-coding RNAs (lncRNAs) in developing and enhancing the tumor phenotypes have been unfolded. This review attempts to shed light on understanding the influence of long non- coding RNAs in the modulation of various CSC-signaling pathways and its impact on the CSCs and tumor properties; highlighting the protagonistic and antagonistic roles of lncRNAs.

## 1. Introduction

Initially entitled as junk matter, non-coding RNAs are an exceptional class of RNAs constituting a majority of the transcriptional output in human cells, which are not translated into functional proteins. They are not only responsible for regulating the expression of the gene at the transcriptional and post-transcriptional stages but also for mediating various cellular processes such as heterochromatin formation, epigenetic modifications, signal transduction, etc. [1]. With advances in research outcomes, it is quite evident that non-coding RNAs (ncRNAs) play a considerable role in modulating various diseases and disorders, especially cancer. A special subpopulation of cancer cells differentiating into heterogeneous cancer cell populations and exhibiting self-renewal ability are recognized as cancer stem cells (CSCs). They direct tumor-initiating, developing, and progressing activities. These tumor-initiating cells also play key roles in distant metastasis, relapse, and cell death, and therapy resistance. Various cell signaling pathways, such as NOTCH, WNT/β-catenin, TGF-β, STAT3, and Hedgehog pathways, play crucial roles in normal stem cell (NSC) homeostasis and work in an interconnected manner to maintain their characteristics. Any abnormality or malfunction in these pathways can influence the characteristics of CSCs, altering and deregulating self-renewing, cell differentiating, and proliferating activities [2]. Substantial reports have indicated that various non-coding RNAs have affected cancer development and progression by operating alone or by functioning in synergy as oncogenes or tumor suppressors [3,4,5]. Recent reports have made evident that these ncRNAs have influenced the hallmarks of cancer by not only acting as tumor suppressors and promoters but also by upregulating and downregulating different CSC-signaling pathways.

## 2. Cancer Stem Cells

About 180 years ago, based on the morphological similarities between tumor cells and embryonic cells, Johannes Muller characterized tumors as the abnormal prolongation of embryonic cell development. He erroneously stated that tumor development does not initiate from the normal tissue cells but from scattered germ cells among normal tissues [6]. Furthermore, Rudolf Virchow highlighted the correlation between tumor and embryonic development, indicating that these processes are outcomes of cell division and multiplication. His student, Julius Cohnheim, proposed that certain persistent embryonic cells act as the common originators of all tumor cells. In the presence of an appropriate microenvironment, these embryonic cells undergo uncontrolled proliferation to eventually form tumor cells [7]. These theories provided stepping stones to the idea of cancer stem cells. At the end of the twentieth century, cancer stem cells were discovered through the contribution of John Dick’s laboratory; additionally, their studies provided evidence on tumor induction by cancer stem cells (leukemia-initiating stem cells). Their studies illustrated that this tumor-initiating activity of human acute myeloid leukemia cells in immunocompromised mice was only exclusive to the CD34+, CD38− leukemia subpopulation. They also demonstrated that these cells depict high self-renewal capacities, which is a crucial stem cell hallmark [8,9]. In the early 2000s, CSCs were identified in solid tumors for the first time in breast cancer. Later, CSCs were discovered in various other solid tumors from the brain [10], liver [11,12], pancreas [13], colon [14], skin [15], bladder [16], stomach [17], lung [18], and head and neck [19].

The origin of CSCs is a debated topic and a discipline in active research. Some of the different major hypotheses attributing to the origin of CSCs are mutational changes in stem cells existing in tissues, differentiating cells attaining stem-cell-like properties, the transformation of adult stem cells and adult progenitor cells, mutational changes in early precursor cells rendering them to reacquire self-renewal properties [20]. CSCs share similarities such as self-renewal and differentiation, regulation of signaling pathways, angiogenesis stimulation, and the expression of similar surface receptors with normal stem cells. They can be differentiated by characteristics such as shorter telomeres, abnormal chromosomal numbers and rearrangements, less mitotically active cells, altered niche or microenvironment. The microenvironment of CSCs plays a vital role in the maintenance of their properties; thus, CSCs regulate their niche in order to promote tumor development, sustain phenotypic plasticity, modulate the immune response, and conserve their characteristics.

**Figure 1:**
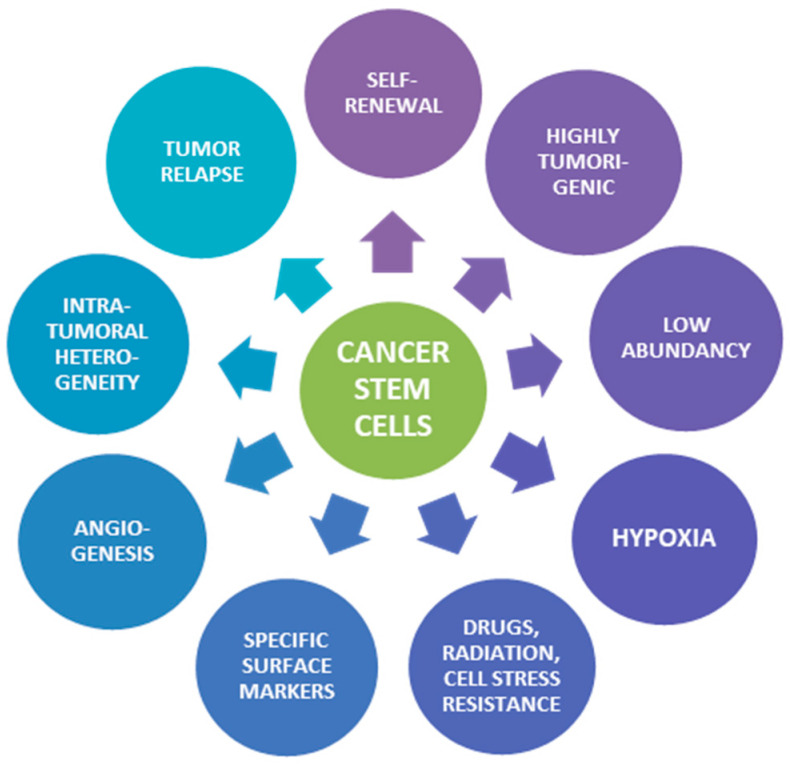
Characteristics of Cancer Stem Cells.

CSC proliferation is stimulated in a hypoxic microenvironment due to elevated expression of hypoxia-inducible factor (HIF) and cancer-associated fibroblasts (CAF); such an environment may instigate necrosis in the tumor mass [21]. Hypoxia can also influence multidrug resistance (MDR) through increased expression of ATP-binding cassette (ABC) transporters leading to a sustained CSC population by decreased drug concentration. Hypoxia and MDR forms one of the major hallmarks of cancer stem cells, others include properties such as self-renewal and differentiation which not only lead to intra-tumoral heterogeneity but also aid in tumor recurrence (Figure 1). They express specific cell surface markers such as CD24, CD34, CD44, CD90, CD133, and CD166, or intracellular markers such as ALDHs, TGF-β, β-Catenin, Nanog, etc. [22]. CSCs stimulate angiogenesis by hypoxia, chemical signals, or pro-angiogenic factors, which in turn elevate CSC survival via the production of growth factors. This facilitates oxygen and nutrient uptake further leading to tumor growth, progression, metastasis, and also recurrence.

## 3. Cell Signaling Pathways Associated with CSCs

An in vivo location that accommodates a certain microenvironment where the stem cells are sustained in an undifferentiated and self-renewable stage is known as a stem cell niche [23]. These niches are found in the different anatomical locations where they administer various cell–cell interactions to regulate the fate of stem cells [24]. The microenvironment participates in the stem cell’s development, proliferation, fate, and interactions with other cells by responding to different stimuli, such as growth factors, secreted cytokines, and different cellular signals. These stimuli can be regulated by several genetic and epigenetic activities [25]. The cell signaling pathways modulating the NSCs and CSCs are similar. These pathways are responsible for strictly regulating the properties of NSCs but this control is lost in the case of CSCs. Various signaling pathways such as NOTCH, WNT, STAT, Hedgehog, TGF-β, IL6, and NF-kB are found to be deregulated in CSCs [26,27]. The deregulation of these homeostatic signaling pathways can encourage unmediated differentiation as well as self-renewal of CSCs which may further enhance its characteristics [28]. This also affects the stimuli interacting with the CSC microenvironment. For instance, cell recruitment can be initiated by the secretion of soluble factors from the primary tumor. Secretion of growth factors such as VEGF, EGF, FGF, HGF, PDGF, TGF-β, and TNF-α from the primary tumor promotes angiogenesis [29,30]. These factors also alter downstream signaling pathways such as NOTCH, WNT/β-Catenin, PI3K/AKT, MAPK/ERK, and TGF-β/SMAD, to activate the epithelial–mesenchymal transition (EMT), which aids the formation of metastatic characteristics by augmenting mobility and invasion, as well as instigating immune suppression, MDR, and circumventing apoptotic mechanisms. In the cancer stem-cell niches, alterations in these pathways can not only affect the self-renewal ability of cancer stem cells but can also initiate and enhance tumor formation and metastasis.

The NOTCH signaling pathway is known to be associated with regulating cell growth, differentiation, cell fate decisions, during embryogenesis and early developmental processes [31]. NOTCH signaling involves communication between the NOTCH ligands and the NOTCH receptors expressed on the surface of the surrounding cells [32]. After binding, the receptor domain is cleaved by γ-secretase into an intracellular domain, which eventually activates the transcription of NOTCH targets. ADAM Metallopeptidase domains 10 and 17 (ADAM10; ADAM17) transact with nuclear factors to modulate the expressions of the target gene that regulates cell differentiation [26]. The pathway has substantial roles in cell-to-cell interactions, tissue differentiation, and stem cell’s self-renewal. In various tumors, this pathway becomes dysfunctional and is associated with the development of tumor stem cells and the induction of EMT phenotypes [33]. Apart from this, it is also involved in the modulation of CSC self-renewal. This regulation depends on the type of NOTCH receptor that is interacting with the tumor cells. For instance, the NOTCH2 receptor strictly mediates the self-renewal of liver CSCs [34,35].

The sonic Hedgehog or Hedgehog pathway is involved in cell growth, cell specialization, and the patterning of the body during early developmental stages [36]. It is also responsible for modulating processes such as cell differentiation, proliferation, and cell polarity mechanisms [37,38]. Mutations in this pathway during the early developmental processes can lead to prenatal disorders. These mutations and alterations can also lead to tumor development and carcinogenesis [18]. In the case of CSCs, this signaling pathway is involved in their sustenance and proliferation. Dysregulation of the pathway in different cancer types, such as hepatic, pancreatic, bladder, and gastrointestinal cancers, is syndicated with the development of EMT and the generation of CSCs [39,40]. In certain highly tumorigenic pancreatic cancers, it is observed that the obstruction of this signaling pathway impairs the self-renewing ability of pancreatic cancer stem-cells (PCSCs) and also reverses chemo-drug resistance [41]. Deregulations in the Hedgehog signaling pathway also affect the CSC microenvironment in a way that either directly or indirectly enhances the CSCs’ properties. In some cancers, the activities of Patched and Smoothened (SMO) receptors of Hedgehog signaling contribute to cancer progression.

The WNT signaling pathway is known to be associated with regulating proliferation, differentiation, cell polarity, fate determination, and neural patterning during early development [42]. This signaling pathway interacts with different stem-cell niches to sustain various stem cells in their self-renewing state. Along with β–Catenin, the pathway instigates stem cell proliferation and elicits differentiation into different lineage-influenced cell types. Dysregulation or deregulation of the pathway can lead to oncogenic activities and, in some cases, even tumor-suppressive effects. Mutations and alterations in adenomatous polyposis coli protein (APC) and/or Axin are observed in a range of cancer types highlighting the deregulation of the WNT pathway. Moreover, abnormal alteration in WNT genes and/or the signaling pathway may lead to an unusual expression of WNT proteins and ligands leading to cancer initiation [43,44,45,46]. This is observed in bone cancer [47], breast cancer [48], non-small cell lung cancer, and hematologic cancers [49]. β-Catenin signaling is crucial in sustaining CSCs’ phenotype and it is observed in human squamous cell carcinoma that its inhibition results in substantially decreased tumor proliferation. Dysfunctional WNT/β-Catenin pathway prompts cancer cell proliferation in acute myeloid leukemia (AML) by switching on the genes coding for specific oncoproteins and cell cycle regulators [50].

A range of signal transducers and activators of transcription (STAT) components participating in the STAT signaling pathway are responsible for modulating somatic cell differentiation, multiplication, apoptosis, as well as immune response [51]. The STAT pathway also plays functional roles in various stem-cell niches. As an example, in hematopoietic stem cells, the stem cell factors, c-Kit and thrombopoietin, that affect the self-renewal abilities of the stem cells are responsible for stimulating the STAT5 protein [52]. Deregulation of the pathway is linked with CSCs’ induction. When STAT proteins are subjected to Janus family tyrosine kinases (JAKs)-induced phosphorylation, they undergo nuclear translocation in order to eventually express the target genes [53]. The JAK-STAT3 signaling pathway induces tumorigenesis through stimulating pre-metastatic niche, inflammation, and stem cells cross-talk [54]. Abnormal processing of STAT signaling is involved in fueling normal cell transformations, oncogenesis, CSC renewal, and tumorigenesis [55,56]. The deregulated STAT pathway can promote stem cell self-renewal but strangely it can also impede cell differentiation pathways [57]. In prostate cancer, it was observed that STAT3 activation induces stem cell-like phenotypes due to regression of androgen receptor expression [58]. The expression of STAT5 is seen to influence hematological malignancies [54]. In breast cancer, the CD44+/CD24− CSC’s self-renewal properties were enhanced due to the synergic effect of epigenetic factors and the activated JAK-STAT pathway [59].

The TGF-β signaling pathway participates in cellular processes during early development as well as during adulthood. These processes include cell growth, differentiation, cell homeostasis, apoptosis throughout embryonic development, and adult tissue homeostasis [60]. TGF-β is involved in differentiation, stemness modulation, tissue repair, and maintenance in stem cells. During carcinogenesis, in some cancer types (e.g., colorectal, pancreatic) loss of function of SMAD complexes is observed which leads to deregulated signaling pathways [61,62,63]. The cross-talk of TGF-β with CSCs causes initiation, development, and progression of various tumors; additionally, it is also responsible for imparting cells with CSC-like properties [64].

The NF-κB signaling pathway is known to modulate certain cellular activities such as proliferation, innate immunity, stress response, and inflammation. It also plays a significant role in sustaining stem cell pluripotency. In a broad range of cancer types, NF-κB signaling is directly linked with various mechanisms of carcinogenesis and tumor progression [65,66,67,68,69]. The canonical pathway is known to regulate crucial cancer-promoting processes such as EMT, angiogenesis, cell proliferation, invasion, metastasis, and evasion of apoptosis. Increased NF-κB activity is seen in niches with high oncogenic mutations and a prolonged inflammatory microenvironment [70,71]. In the case of colon cancer microenvironments with chronic inflammation, it was observed that due to escalated NF-κB activity there was an accumulation of pro-inflammatory cytokine mutations. These accumulations in turn stimulated pro-tumorigenic properties in the microenvironment [72]. NF-κB also functions as an anti-inflammatory agent during an immune response, which aids in MDR and tumor development [73]. For example, NF-κBp50 overexpression in cancer-related M1 macrophages was seen to have an obstructing effect on antitumor and inflammatory reactions in murine fibrosarcoma, and similar activity was also seen in human ovarian carcinoma [74]. In the early stages of tumorigenesis, the expression of various oncoproteins such as mutant RAS and BCR-ABL causes NF-κB induction. This causes cancer initiation, recurrence, and proliferation. This pathway drives the regulation of crucial target genes of EMT transcription factors, IAPs (inhibitors of apoptosis), cytokines, and CSC phenotypes [74].

## 4. Non-Coding RNAs and Their Interaction with CSCs

From the past decade, it has become evident that the non-coding portion of the human genome is not mere transcriptional noise, as originally believed. Rising proof shows that the previously declared “dark matter” of the genome may play a crucial role in our health and disorders. Non-coding RNAs are divided into small ncRNAs, mid-size ncRNAs, and long ncRNAs, ranging from 18–30, 50–200, and > 200 nucleotides in length, respectively. They are responsible for regulating the expression of the gene at the transcriptional and post-transcriptional stages as well as for mediating various cellular processes such as heterochromatin formation, epigenetic modifications, signal transduction, etc. Non-coding RNAs play a considerable role in modulating various diseases and disorders, especially in cancer. Various ncRNAs have affected cancer development and progression by promoting hallmarks of cancer as oncogenes or antagonizing them as tumor suppressors. From recent studies, it is evident that these ncRNAs have influenced cancer cells and tumor microenvironments by upregulating and downregulating different CSC signaling pathways.

MicroRNAs are one of the common ncRNAs which are connected in the regulation of a wide range of cancer processes, such as transformation, cancer cell proliferation, EMT, invasion, and metastasis. They work by hindering the expression of crucial genes in various pathways that modulate cellular activities including cell cycle, apoptosis evasion, and cell migration [75,76].

Recent reports have also shown that other small non-coding RNAs are also involved in regulating various cancer-linked processes as well as in modulating different CSC phenotypes. For instance, piRNA-651 was found to be upregulated in the lung, gastric, breast, liver, mesothelial, and cervical cancer cell lines [77]. The repression of this sncRNA by antisense oligonucleotides caused a highly decreased proliferation in gastric cancer cells and cycle arrest (G2/M phase). Contrastingly, another sncRNA, piRNA-823, was substantially lowered in gastric cancer tissues. More and more pieces of evidence suggest that the deregulation of other ncRNAs, such as piRNAs, siRNAs, and snoRNAs, is also associated with tumor generation, development, and metastasis [78].

## 5. Focus on LncRNAs

One of the types of non-coding RNAs that span higher than 200 nucleotides in length, possess no mechanism to code for proteins, and are seen to have a little expression in certain tissues are considered as long non-coding RNAs. Some lncRNAs may show exceptions to these criteria. It is quite evident from the recent research that the abnormal expression of LncRNAs plays a significant role in CSCs’ metabolism. They regulate gene expression by the following approaches: as a modulator of gene expression; as a decoy to lead the transcription factor elsewhere from a target site; as a competitor to hinder the attachment of other molecules to the target site; as a chaperone for molecules to attach to a certain segment and as a scaffold that enhances the association of different proteins into different complexes [79].

Different types of lncRNAs regulate the functions of CSCs by altering and modulating different transcription factors, enzymes, niches, and signaling pathways. LncRNA H19 is seen to maintain the stemness in glioma [80] and BCSCs [81]. Contrastingly, lncRNA ROR plays a repressive role in glioma SCs by negatively altering the expressions of stem cell factor KLF4 [82]. LncRNA CUDR leads to high H3K4 trimethylation by activating the associations between SET1A and pRB1in hepatocellular carcinoma (HCC) [83]. It works with Cyclin-D to stimulate the self-renewal and proliferation of hepatocellular carcinoma stem cells by increasing the expression of telomerase reverse transcriptase (TERT) and C-Myc [84]. In prostate cancer, the PCR2-LncRNA HOTAIR association represses the androgen receptor transcription by complexing to its 5′-flanking promoter region and enhancing prostate cancer stem/progenitor cells and invasion [85]. In breast cancer, LncRNA HOTAIR leads to the induction of EMT by underregulating the STAT3 pathway [86]. LncRNA uc.283-plus [87], LncRNA CRNDE [88], and LncRNA XIST [89] are all known to be overregulated in glioma stem cells. Two lncRNAs, PRNCR1 and PCGEM1, are found to be overexpressed in different prostate cancers and cause the androgen receptor (AR)–associated transcriptional mechanisms to induce the growth of prostate cancer [90,91]. Maternally expressed gene 3 lncRNA (MEG3) was observed to stimulate p53 and promote p53 signaling, as well as increasing p53 associations with the promoters of its target genes [92]. Recurrent hypermethylation in the MEG3 promoter is commonly seen in human tumors, such as pituitary cancer [93], meningioma [94], and leukemia [95]. Higher expression of MEG3 works in a tumor suppressive manner by oppressing cell proliferation in meningioma and HCC cell lines [96].

## 6. The Interplay of LncRNAs in CSCs Signaling Pathways

### 6.1. NOTCH Signaling Pathway

NOTCH signaling is a majorly conserved signaling pathway that carries out juxtracrine signaling between cells. NOTCH signaling takes place by the binding of two jagged ligands (Jagged1; Jagged2) and a transmembrane delta-like ligand either 1, 2, 3, or 4 (DLL1, DLL2, DLL3, DLL4) and one cell to a NOTCH receptor either 1, 2, 3, or 4 (NOTCH1, NOTCH2, NOTCH3, NOTCH4) on the neighboring cell. In the endoplasmic reticulum, NOTCH is synthesized and glycosylated by the soluble ER enzymes. In the Golgi, the S1 cleavage generates a heterodimer of NOTCH extracellular domain (NECD) and transmembrane NOTCH intracellular domain (TM-NICD). It then is transported to the plasma membrane to permit ligand binding. This binding is modulated by Deltex and obstructed by the activity of NUMB. After this, the S2 cleavage takes place, the NECD is cleaved from the TM-NICD domain by TNF-α ADAM metalloprotease converting enzyme (TACE) ADAM17 or a disintegrin and metalloproteinase domain-10 (ADAM10). In the signal-sending cell, the NECD stays confined to the ligand and experiences further recycling. Meanwhile, in the signal-sending cell, S3 cleavage is carried out by γ-Secretase, which releases the NICD segment. It is then translocated to the nucleus, where it associates with transcriptional regulators to instigate transcription of the NOTCH target genes.

NOTCH signaling plays a prominent role in the modulation of cell-fate specification, and progenitor cell differentiation, maintenance, and proliferation during organogenesis as well as during the early development of the hematopoietic cells and Central Nervous System [97,98]. In stem cell niches, it is crucial in the differential activities of some stem cells, stem cell proliferation, and their interaction with the microenvironment. The carcinogenicity of the pathway was first observed in human T-cell acute lymphoblastic leukemia (T-ALL), where, due to the release of N1ICD, NOTCH-1 was seen to be activated [99]. Since then, abnormal activation of the NOTCH family led to tumorigenesis of various cancers, such as pancreatic [100], breast [101], and cervical cancer [102]. This pathway is observed to operate as both oncogenic and tumor-suppressive, this is dependent on the tissue type, cancer cell type, as well as the stage involved. NOTCH-1 stimulates cancer development at an early stage of cervical cancer, while it represses cancer growth at a later stage. As an oncogene, NOTCH is seen to be overexpressed in T-ALL, pancreatic, breast, gastric, and colon cancer [100,101,103,104,105]. As an example, mutations leading to activated NOTCH1 can stimulate the development of T-ALL [106]. PCSCs were observed to have increased levels of NOTCH1, NOTCH3, Jag1, Jag2, as well as the NOTCH target gene Hes1; this was responsible for the maintenance of CSCs stemness [107]. Meanwhile, its expression is downregulated in myeloid malignancies, skin, non-small cell lung cancer, liver, prostate, and certain breast cancers [108,109,110,111,112] where it plays a tumor-suppressive role. It was observed that NOTCH-1 activation is responsible for inducing the MMP-2 and MMP-9 expression and stimulation while lowering the regulation of NOTCH-1 involved in lowering the MMP-2 and MMP-9 activation to hinder cell progression in breast cancer and pancreatic cancer cells [113].

**Figure 2:**
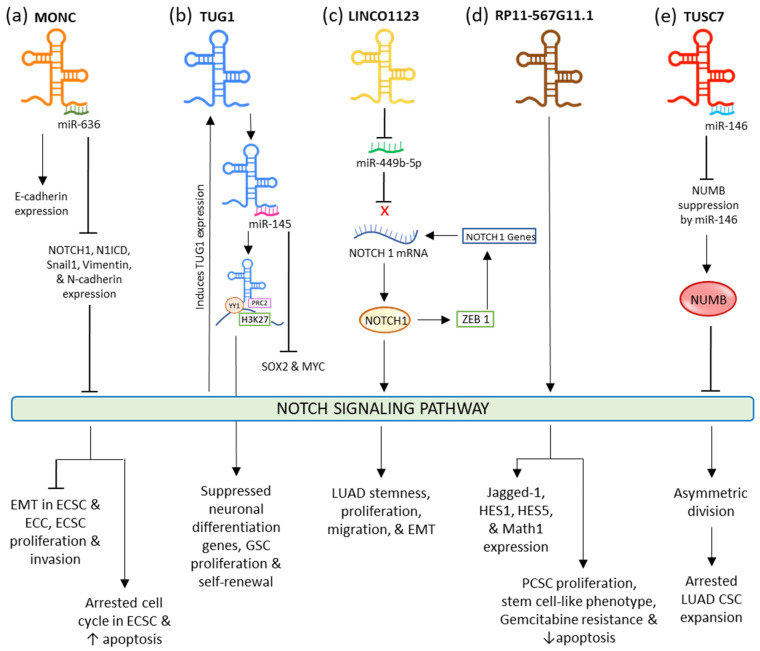
Interaction of different lncRNAs and lincRNA with NOTCH signaling pathway.

In endometrial carcinoma (EC), tumor-suppressive MONC sponges oncogenic miR-636 to negatively regulate it. The upregulation of this lncRNA restricted the expression of NOTCH1, N1ICD, Vimentin, Snail1, and N-Cadherin, while the expression of E-Cadherin was fostered. This eventually inhibited the NOTCH signaling pathway and EMT resulting in suppressed proliferation and invasion, and induced apoptosis, and aided the arrest of the cell cycle at the G0/G1 phase in endometrial cancer cells and cancer stem cells (ECCs and ECSCs) (Figure 2a) [114]. Increased expression of lncRNA TUG1 (Taurine Upregulated Gene 1) is seen in different types of cancers such as bladder carcinoma, stomach cancer, and bone sarcoma [115,116,117], while its decreased expression is seen in non-small cell lung cancer (NSCLC) [118]. This lncRNA is credible for sustaining the stemness properties and proliferation of glioma stem cells (GSC) in glioblastoma via neutralizing miR-145 by sponging it in the cytoplasm and modulating transcription factors MYC and SOX2. In the nucleus, it forms a polycomb structure with YY1, PRC2, and H3K27, from which histone H3K27 undergoes locus-specific methylation via the activity of YY1 to limit the neuronal differentiation genes (Figure 2b). In lung adenocarcinoma (LUAD), ZEB1, an EMT transcriptional factor, activates LINC01123 and NOTCH1. LINC01123 functions by sponging miR-449b-5p and blocking its inhibitory effect on NOTCH1 mRNA; this results in the expression of NOTCH1, thus mediating the promotion of the NOTCH1-dependent NOTCH signaling pathway. This results in the lncRNA facilitating increased stemness, proliferation, migration, malignant transformation, and EMT in LUAD (Figure 2c) [119]. In pancreatic cancer, RP11-567G11.1 is observed to upregulate the NOTCH signaling pathway and the expression of its downstream components. This aberrant activation resulted in the progression of the cell cycle, PCSCs proliferation, tumorigenesis, and decreased apoptosis. RP11-567G11.1 also mediated the chemoresistance to antimetabolite Gemcitabine (Figure 2d) [120]. LncRNA TUSC-7 is responsible for inactivated NOTCH signaling in lung adenocarcinoma (LUAD) stem cells. This leads to the disruption in the symmetric division, causing the appointment of asymmetric division and ultimately arresting the CSC expansion.

It does so by inactivating the miR-146-mediated repression of NUMB, which plays a significant role in restraining the NOTCH signaling. It is responsible for restraining the ligand from binding by hindering the NOTCH receptor’s endosome transportation to the plasma membrane. When TUSC-7 is sponged to miR-146, its degradation toward NUMB is abolished, resulting in the inactivation of NOTCH signaling (Figure 2e) [121].

### 6.2. WNT Signaling Pathway

WNT signaling significantly participates in different biological activities, such as cell proliferation, differentiation, tissue regeneration, and organogenesis [122,123,124,125,126]. This pathway is split into β-Catenin-dependent and β-Catenin-independent signaling; the former is known as the canonical pathway which is the WNT/β-Catenin signaling pathway and the latter is known as the non-canonical pathways comprised of WNT/calcium signaling and WNT/planar cell polarity [PCP]-signaling pathways [127,128].

The canonical WNT signaling pathway is initiated when WNT-associated ligands link to Frizzled, a transmembrane receptor and the low-density lipoprotein receptor-related protein (LRP). Phosphorylation of LRP is then carried out, due to which Dishevelled proteins (DVL) become polymerized at the plasma membrane. This further hinders the destruction complex; comprising APC, AXIN, and GSK3β. This leads to the accumulation of β-Catenin in the cytoplasm, which then undergoes nuclear translocation. Once inside, β-Catenin is associated with the T-cell factor (TCF) and lymphoid enhancer factor (LEF); it functions by regulating multiple cellular mechanisms, thus behaving as a transcriptional switch [129].

In the non-canonical calcium-signaling pathway, WNT binds to DVL to stimulate phospholipase C (PLC) which releases calcium ions. These intracellular ions stimulate the downstream protein kinase C (PKC) and calcium/calmodulin-dependent protein kinase II (CaMKII) which in turn positively affect the nuclear factor (NFAT) [130]. In the non-canonical PCP-signaling pathway, the planar cell polarity is triggered by WNT–Frizzled receptor associations that activate DVL. Activated DVL then modulates RHOA, RAC, and CDC42 GTPases. It further triggers the JUN N-terminal kinase, which then activates nuclear factor-dependent transcription of activator protein-1 and stimulated T cells after nuclear transfer.

The canonical pathway is mostly known as regulating cell proliferation, while noncanonical pathways manage cells’ polarity and movement. However, both signaling pathways participate in tumorigenesis and can also become involved in different cellular processes [131]. In the same way, adenomatous polyposis coli (APC) and β-Catenin not only regulate cell proliferation but are also involved in cell-to-cell adhesion [132]. In colon cancer, R-SPONDIN/LGR5/RNF43 mutations are linked with the deregulation of WNT leading to tumor development [129]. Approximately 92% of sporadic colorectal cancers are observed to have at least an alteration in the regulators of the WNT pathway [133]. In a wide range of breast cancers, plummeted levels of nuclear β-Catenin are reported, as well as seeing regular plummeted expression of the receptors and ligands. However, unlike colorectal cancer, the development of breast cancer is not due to the genetic regulation of the signaling components of the WNT pathway, but is due to other factors such as epigenetics [134]. In different types of leukemias, the deregulated WNT pathway leads to increased WNT activity, eventually triggering leukemogenesis. In liver-cancer stem cells (LCSC), activation of canonical WNT signaling is linked with elevated self-renewal capacity of cells [135]. WNT signaling was seen to elevate the expression of a colon cancer SC marker CD44v6, which resulted in an aggravated metastatic capacity of the CSCs [136]. In the breast cancer niche, the cross talk of Periostin with WNT1 and WNT3a stimulates WNT signaling, resulting in the sustained phenotype of cancer stem cells [137].

**Figure 3:**
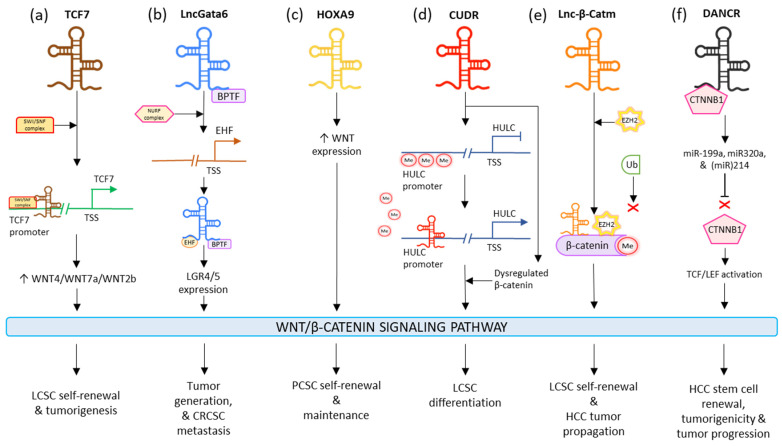
Influence of different lncRNAs on WNT/β-Catenin CSC signaling pathway.

LncTCF7 appoints the SWI/SNF to the TCF7 promoter and improves its expression to surge the levels of WNT7a/WNT4/WNT2b, which activates the WNT signaling pathway. The lncTCF7-associated WNT stimulation results in the maintenance of the self-renewal and tumorigenic ability of LCSCs (Figure 3a) [135]. LncGata6 enhances tumor generation and metastasis in colorectal CSCs. LncGata6 associates with Bromodomain PHD Finger Transcription Factor (BPTF) and adopts the Nucleosome Remodeling Factor (NURF) complex to trigger ETS homologous factor (EHF) transcription, which makes up the lncGata6–BPTF–EHF axis to generate LGR4/5 expression and eventually activate the WNT signaling pathway (Figure 3b) [138]. LncRNA HOTTIP is observed to drive the stimulation of the WNT/β-Catenin signaling pathway; this induces the cell generation and chemoresistance in osteosarcoma. It is also responsible for elevating the stemness of PCSCs by regulating the WNT pathway. HOXA9 regulates the stimulation of the WNT pathway in PCSCs by promoting WNT. HOXA9 targeted stem cell transcription factors (SOX2, Nanog, OCT4, and LIN28) and markers (ALDH1, CD44, and CD133) in order to sustain PCSC properties such as inducing CSC expansion and self-renewal (Figure 3c) [139]. Interestingly, LncRNA CUDR stimulates lncRNA HULC by obstructing the methylation of the HULC promoter; additionally, it dysregulates β-Catenin thus promoting malignant differentiation in human-liver stem cells (Figure 3d) [140]. LincRNA-p21 is seen to reduce the activity of β-Catenin signaling, ultimately mediating the negative regulation of self-renewal, viability, and glycolysis of colorectal-cancer stem cells (CRCSCs) [141]. The levels of lncRNA H19 are observed to plummet in bladder cancer tissues. H19 is complexed with the enhancer of zeste homolog 2 (EZH2), such that this association results in the activation of WNT/β-Catenin. This activation reduces the expression of E-cad and increases tumor metastasis. Upregulated H19 is also responsible for promoting bladder-cancer cell migration [142]. Lnc-β-Catm catalyzes the methylation process of β-Catenin, by binding to it along with methyltransferase EZH2. This limits the ubiquitination of β-Catenin and thereby stabilizes it, which eventually drives the activation of the WNT/β-Catenin signaling pathway. This activation promotes self-renewal and maintenance of LCSCs and tumor progression of HCC (Figure 3e) [143]. In HCC, microRNAs miR214, miR320a, and miR-199a are seen to have a suppressive effect on CTNNB1; DANCR blocks this suppression by associating with tethering to CTNNB1. This induces the activation of TCF/LEF by β-Catenin, which guides the positive regulation of HCC stem cells’ self-renewal, tumorigenicity, and cancer progression (Figure 3f) [144]. In gastric cancer, HOTAIR was seen to be majorly regulated in cisplatin-resistant gastric cancer cells and tissues. It was observed that the higher expression of HOTAIR promoted gastric-cancer cell proliferation, enhanced the transition of cell cycle G1/S, as well as decreased cancer cell apoptosis by activating WNT/β-Catenin signaling. Moreover, the lncRNA was also responsible for binding and inhibiting miR-126 expression; this resulted in the promotion of VEGFA and PIK3R2 expression and activation of the PI3K/AKT/MRP1 pathway [145].

## 7. STAT Signaling Pathway

In humans, the STAT family incorporates STAT-1, 2, 3, 4, 5A, 5B, and 6 [146]. Among these seven proteins, STAT-3 and STAT-5 demonstrate the highest association with tumor progression. Their activation regulates a broad range of functions, such as cell cycle progression, proliferation, apoptosis, angiogenesis, as well as immune evasion [147,148,149]. STAT-3 is primarily responsible for tumor growth, proliferation, and sustenance due to its activity in stromal cells, immune cells, and the tumor microenvironment [150,151,152,153,154,155]. The negative modulation of STAT-3 activation is carried out by protein repressors of stimulated STATs (PIAS), tyrosine phosphatases, and oppressors of cytokine signaling (SOCS) [156].

Abnormal activation of the pathway is seen in CSCs from different cancers such as breast, blood, glia, and prostate. In prostate cancer, stem-like cells were seen to overexpress various genes linked to JAK/STAT signaling pathway, such as IFNK, IFNGR, IL6, CSF2, and STAT1 [157], and stimulate STAT3 to prominently regulate JAK/STAT signaling molecules in mammalian CSC-like cells [158]. In glioblastoma, obstruction of STAT3 in CSCs shuts down proliferation, as well as lowering the expression of OLIG2 and NESTIN (neural stem cell gene), and enhancing the expression of βIII-Tubulin, which acts as a neuronal differentiator [159]. Thus, in glioblastoma, JAK/STAT signaling is significant for CSC proliferation and is responsible for positively regulating glioblastoma stemness. In acute myeloid leukemia (AML), activated JAK/STAT signaling led to the growth and sustenance of CSCs [160].

Abnormal STAT3 activation, because of tyrosine 705 phosphorylation signaling, led to the oncogenesis and induction of malignancy [161,162,163,164]. Activation of STAT3 in the tumor causes transfer of indicators from the different growth factors and cytokines [165] which in turn induces certain target genes such as Cyclin-D, c-Myc, and CDC25A. These factors ultimately activate the proliferation of cells, suppress the apoptotic genes [166], and upregulate anti-apoptotic genes such as Beta2-Macroglobulin, B-Cell CLL/Lymphoma-2 (BCL2), and BCLXL [167]. The induction of STAT3 by IL-6 [168] or stress factors [169] leads to progress in the self-renewal of prostate CSCs and tumor-propagation [170]. VEGF binds to VEGFR-2/JAK2/STAT3, leading to the activation of STAT3 and upregulation of Myc and SOX2. This eventually results in enhanced self-renewal of breast and lung CSC. Thus, apart from angiogenesis, VEGF is also responsible for CSCs’ self-renewal and tumor-initiating via the VEGFR-2/STAT3 signaling [171].

**Figure 4:**
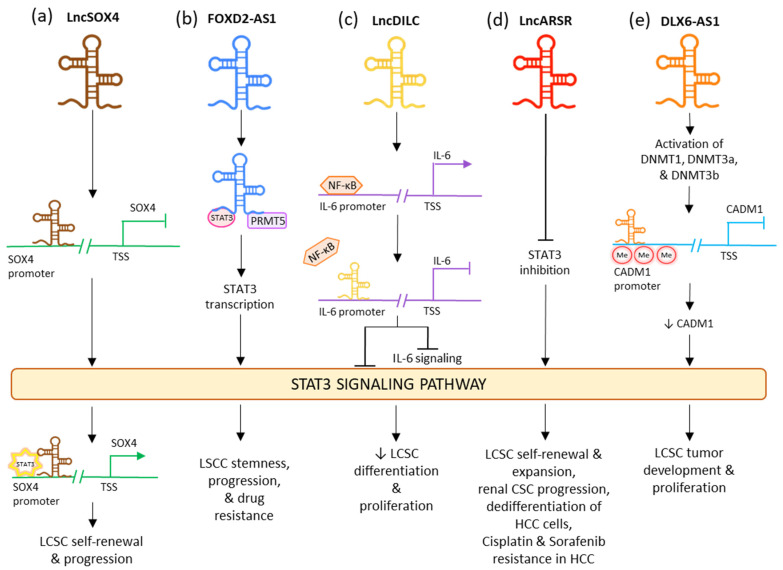
Influence of different lncRNAs on STAT3 CSC signaling pathway.

LncRNA downregulated in liver-cancer stem cell (lnc-DILC) acts as a tumor suppressor by declining the proliferation rates of CSC and differentiation of the LCSCs [172]. It also has an intense hindering effect on the autocrine pathway of IL-6/JAK2/STAT3 signaling [173]. This lncRNA lowers the expression of STAT3 as well as IL-6 and also limits the translocation of STAT3 to the nucleus. It also acts by inhibiting the NF-κB-mediated induction of IL-6. In various tumor stem cells, IL-6 is highly expressed by the NF-κB signaling pathway which is triggered by growth factors such as TNF-α and IL-1β (Figure 4c) [174,175]. LncRNA DILC and lncSOX4 are well known for controlling CSC properties by the STAT3 pathway in liver cancer. Lnc-DILC can also regulate the JAK2/STAT3 signaling. Overexpression of lnc-DILC lowers the elevated levels of phospho-STAT3 protein, reduces the translocation of STAT3 to the nucleus, and oppresses the transcriptional activity of STAT3-responsive segments. In CSC spheroids, the decreased regulation of lnc-DILC activates TNF-α and IL-1β-triggered IL-6 expression. Thus, this lncRNA can synchronize the crosstalk between the inflammatory signaling and the autocrine IL-6/STAT3 pathway to affect the expansion of LCSC [176]. On the other hand, lncSOX4 increased regulation has been associated with critical liver cancer. It is positively regulated in the CD133+ liver cancer cell population and CSC spheroids and is crucial for the self-renewing activities and tumorigenic capability of the liver CSCs. LncSOX4 crosstalks with STAT3 and inducts it to the promoter region of SOX4, stimulating the H3K4me3 and H3K27ac alterations to increase the SOX4 promoter stimulation and thus SOX4 expression. The lncSOX4/STAT3-dependent SOX4 expression is significant for sustaining liver CSC proliferation (Figure 4a) [177]. Upregulated FOXD2-AS1 was identified in maintaining the stemness in laryngeal squamous cell carcinoma (LSCC) and decreasing the reactions to chemotherapy drugs, while its under-regulation had opposing consequences. FOXD2-AS1 is complexed as a scaffold with STAT3 and PRMT5, stimulating the transcription STAT3, which is important for maintaining tumor stemness and enabling chemotherapeutic resistance. Thus, FOXD2-AS1 acts as an upstream activator of STAT3 and aids cancer progression (Figure 4b) [178]. LncARSR overexpression is seen in CD24+/CD133+ LCSCs as well as in hepatoma spheres augmented with CSC. Its upregulation causes blockage of STAT3 inhibition, thus activating the STAT3 signaling. STAT3 also plays a downstream role in regulation by lncARSR in HCC cells. LncARSR is responsible for LCSC expansion by rising the dedifferentiation of hepatoma cells and increasing the self-renewal, progression, and tumorigenesis capacity of LCSCs. It also aids drug resistance and tumor recurrence (Figure 4d) [179]. In early LSCC, the levels of lncRNA PTCSC3 in plasma are low. The increased expression of PTCSC3 causes repression of LSCC cell proliferation; thus, the lncRNA acts as a tumor suppressor. In UM-SCC-17A cells, PTCSC3 hindered STAT3 and HOTAIR’s expression, while STAT3 was responsible for increasing the expression of HOTAIR. Thus, it can be stipulated that the oppressive effect of PTCSC3 on tumor progression can be connected to lncRNA HOTAIR [178]. The downregulation of LncRNA DLX6-AS1 may cause an inhibitory effect on the methylation of the CADM1 promoter. This may lead to the inactivation of the STAT3 signaling pathway. Upregulated CADM1 results in the suppression of the stem cell characteristics of liver CSCs [180]. In HCC, overactivated lncRNA DLX6-AS1 confers the methylation of CADM1 promoter via activation of methyltransferases DNMTs and expression of SOX4 in LCSCs and thus upregulating the STAT3 signaling pathway. This led to decreased expressions of SOX2, OCT-4, CD13, and CD133 in LCSCs and induced tumor formation and proliferation (Figure 4e) [180].

## 8. Hedgehog Signaling Pathway

Abnormal induction of Hedgehog is linked with the proliferation of cancer cells, neoplastic transformations, malignancy, metastasis, and multi-drug resistance of a wide range of tumors. When Hedgehog is not present, PTCH1 localizes in the cilia and avoids the membrane localization and stimulation of SMO. SMO after internalization undergoes degradation. The full-length GLI (GLIFL) is altered by protein kinase A (PKA), glycogen synthase kinase-3 (GSK3), casein kinase 1 (CK1), and the E3 ubiquitin ligase β-TrCP. It then undergoes cleavage to form transcriptional oppressor GLIR. The stimulated form of GLI (GLIA) is repressed by SUFU. Then, GLIR undergoes nuclear translocation and hinders the expression of its target genes. When the Hedgehog binds to PTCH1, it internalizes and carries out the translocation and induction of SMO. GLIFL avoids phosphorylation by PKA, GSK3, and CK1, which causes activation of GLI (GLIA). GLIA then undergoes nuclear translocation where it activates the expression of Hedgehog target genes [181].

The activated Hedgehog pathway is involved in the growth, survival, migration, and proliferation [182] of a wide range of cancers such as multiple myeloma, pancreatic adenocarcinoma [183], breast cancer [184], and chronic myelogenous leukemia (CML) [185]. The first evidence of deregulated Hedgehog signaling and cancer was seen in Gorlin syndrome, which is caused due to the inducing mutation in the Patched gene [186]. Patients with this syndrome display different cancer types such as basal cell carcinomas (BCCs) [187], medulloblastoma [188,189], and rhabdomyosarcoma [190,191]. The paracrine stimulation of the Hedgehog pathway is seen in different tumor types such as prostate [192], pancreatic [193], colorectal, and esophageal cancers [194]. In chronic myelogenous leukemia (CML), the loss of SMO impaired the self-renewal capacity of the hematopoietic stem cells and hindered the initiation of CML by the oncoprotein: BCR-ABL1 [195]. Sonic Hedgehog, SMO, and GLI1 are highly aggravated in CML suggesting that activation of the Hedgehog pathway is a significant activator of CML progression [196]. Hedgehog inactivation in MCF-7-derived CSCs caused a reduction in the cells via the downregulation of OCT4, NESTIN, and Nanog, suggesting that Hedgehog signaling in breast CSCs upregulates stem cell markers to retain a self-renewing phenotype [197]. This signaling plays a crucial role in the sustenance of colon CD133+ CSCs, which show the increased gene expression levels of GLI1, PTCH1, GLI2, SHH, and HHIP concerning all CD133- cells in colon carcinoma [198].

**Figure 5:**
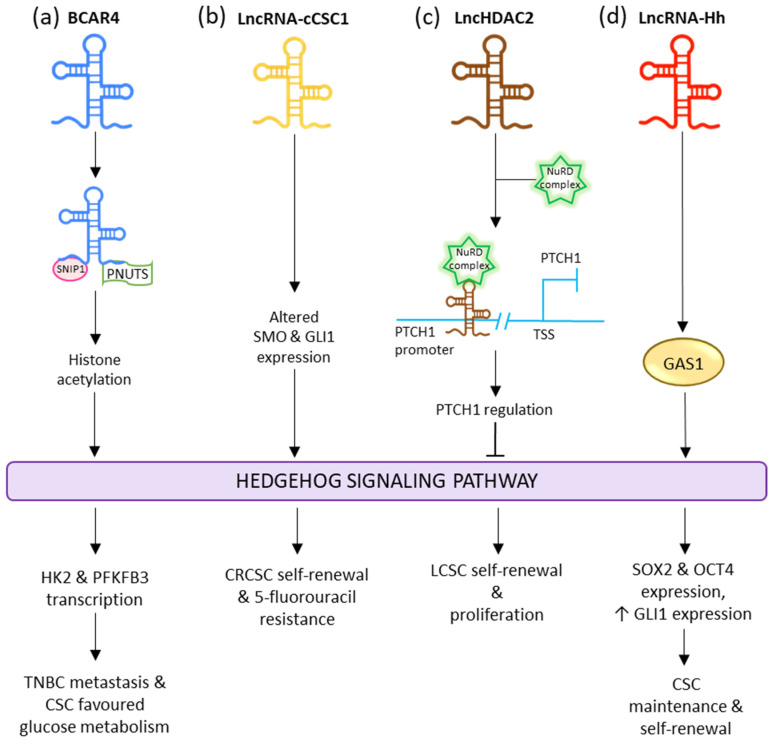
Influence of different lncRNAs on Hedgehog CSC signaling pathway.

Yes-associated protein 1 (YAP) is responsible for upregulating the transcription of lncRNA BCAR4, which interacts with SNIP1 and PNUTS to regulate the p300-dependent histone acetylation carried by H3K27ac of the non-canonical Hedgehog and GLI2 transcriptional cascade. This stimulates the transcription of enzymes HK2 and PFKFB3; thus, allowing BCAR4 to promote metastasis and invasion in triple-negative breast cancer as well as reprogram glucose metabolism in favor of the CSCs (Figure 5a) [199]. LncRNA-cCSC1 is seen to be overexpressed in colorectal cancer cells (CRC) and CRCSCs. The exhaustion of lncRNA-cCSC1 prominently hindered the self-renewal activity of the CRCSCs and decreased their chemo-drug resistance to 5-fluorouracil. Meanwhile increased expression of lncRNA-cCSC1 led to triggered self-renewal of CSCs as well as assisting in 5-fluorouracil drug resistance. Abnormal expression of this lncRNA resulted in the transfiguration of SMO and GLI family zinc finger 1 (GLI1) expression in the Hedgehog signaling pathway (Figure 5b) [200]. LncRNA lncHDAC2 is overexpressed in LCSCs. In the LCSC niche, it is associated with histone deacetylase-2 (HDAC2). LncHDAC2 regulated PTCH1 downregulation to stimulate Hedgehog signaling, thus increasing the self-renewal activity of LCSCs (Figure 5c) [201]. Overexpression of ASAP1-IT1 is seen in cholangiocarcinoma (CCA) tissues and cells. This lncRNA is responsible for activating the Hedgehog pathway via the increased regulation of SMO and GLI1, thus inducing cell proliferation, cell migration, and the progression of EMT [202].

LncRNA EGOT acts as a significant modulator in breast cancer. In gastric cancer, with elevated expression, it acts as an oncogene. EGOT-regulated Cyclin D1 expression in GC is modulated by the Hedgehog pathway. Its upregulation is related to gastric cancer cell growth, cell cycle progression, and lymphatic metastasis [203]. LncRNA HCG18 participates as an oncogenic lncRNA in nasopharyngeal cancer (NPC) progression. It does so by modulating WNT/β-Catenin signaling and the Hedgehog pathway. Upregulation of HCG18 is associated with tumor progressive effects via the stimulation of cell proliferation and metastasis [204]. LncRNA-Hh is known to target GAS1 to induce the activation of Hedgehog signaling. The activated Hedgehog signaling raises the expression of GLI1 and stimulates the expression of SOX2 and OCT4 to regulate CSC maintenance and self-renewal (Figure 5d) [205].

## 9. Other Signaling Pathways

The influence of the long non-coding RNA is not only limited to the above-mentioned pathways but can also be seen in other cancer stem-cell signaling pathways. The PI3K/AKT/mTOR signaling pathway can be regarded as a crucial regulator of cancer stem cells due to its oncogenic role. Activated PI3K triggers phosphorylation of Protein kinase B to activate it, which further mediates mTOR activation. This pathway is upregulated in the majority of cancers, which can have several downstream effects on cancer cells leading to aggravated growth and proliferation, and reduced apoptosis. The association of lncRNA-MALAT1 with miR-124-activated PI3K/AKT signaling leading to induced and promoted HCC CSC generation mediated by Hepatitis-B virus X protein [206]. In glioblastoma, lncRNA SNHG20 positively regulated the PI3K/AKT/mTOR pathway by elevating the levels of p-PI3K, p-AKT, and p-mTOR. This led to decreased apoptosis and increased stemness, proliferation, and tumorigenesis [207]. In osteosarcoma, lncRNA DANCR operated as a post-transcriptional regulator by blocking miR-33a-5p to overexpress tyrosine kinase receptor AXL. This led to the activation of the AXL-mediated PI3K/AKT/mTOR signaling pathway, thus allowing DANCR to promote stemness, proliferation, migration, and invasion [207].

The PI3K/AKT/mTOR signaling pathway is antagonized by PTEN and various other regulators. PTEN functions by dephosphorylating PIP3 to PIP2 and eventually inhibiting the AKT pathway by limiting the activity of AKT. The absence of PTEN not only facilitates the AKT pathway but also other oncogenic processes. In liver cancer, with the increase in scarcity of PTEN in cells, lncRNA CUDR showed enhanced binding to CyclinD1 forming CUDR-CyclinD1 complex. This complex was then recruited by Insulator CTCF, to form the CUDR-CyclinD1-insulator CTCF complex, which further underwent downstream interactions to ultimately increase c-MYC and TERT levels. This resulted in the malignant transformation of liver stem cells and enhanced growth and proliferation of liver cancer stem cells [84].

The MAPK/ERK pathway is observed to mediate the regulation of cellular processes involving growth, differentiation, proliferation, survival, apoptosis, and stress responses, along with gene expression. Thus, dysregulations in this pathway can result in abnormal and uncontrolled functioning of these processes. In various cancer types, MAPK and ERK signals are responsible for driving resistance to chemotherapeutic drugs. In HCC, H19 was found to influence oxidative stress such that it activated MAPK cascades and ERK1/2 through the enrichment of reactive oxygen species in CD133+ liver cancer stem cells. Activation of the MAPK/ERK signaling led the CD133+ LCSCs to acquire resistance to chemotherapeutic agents [208].

The majority of the genes associated with the evolutionarily conserved Hippo-signaling function as tumor suppressors, apart from YAP/TAZ as this transcriptional co-activator is recognized as an oncogene. Its aberrant activation can lead to unregulated growth and malignant transformations. LncRNA NEAT1 was seen to influence the LCSCs by abnormally driving the Hippo signaling pathway. LncRNA THOR positively regulated the TGF-β/SMAD-THOR-β-Catenin signaling pathway by upregulating the expression of β-Catenin. This stimulated the expansion of LCSCs and activated resistance of chemotherapeutic drug Sorafenib in HCC cells [209].

## 10. Impact of Mutations on LncRNA Interaction

With emerging studies as reported above, it is evident that an increasing number of lncRNAs can influence CSCs and different hallmarks of cancer by interacting with classic CSC-signaling pathways. Such interactions of lncRNAs can take a detrimental turn if the integrity of the transcripts is affected by mutations. These RNAs may also be prone to more mutations due to the higher number of nucleotides in their transcriptomic makeup. Apart from CNV and SNP (Single Nucleotide Polymorphism), mutations in lncRNAs can also arise from chromosomal aberration and epigenetic influences, which may impact lncRNA expression and regulation by altering its structure. Moreover, a non-conservative missense mutation in any molecule directly or indirectly involved in lncRNA’s upstream or downstream interactions may either hinder or accelerate its subsequent effects on the tumor’s fate [210]; for instance, mutated molecules or mutations specifically on the lncRNA binding sites of such molecules such as miRNA, transcription factors, regulatory elements, signaling components, etc.

MVIH lncRNA is reported to have an increased expression in hepatoblastoma and HCC cells, causing cancer development and metastasis by enhancing angiogenesis. Interestingly, a tumor suppressor gene ARID1A reduces such proliferative effects by inhibiting the lncRNA expression and positively regulating CDKN1A; its downstream target. However, in a tumor environment with mutated ARID1A, the MVIH inhibition is lifted, affecting CDKN1A expression and ultimately leading to tumor progression [211]. The p53-R273 is observed to regulate around 40 differentially expressed lncRNAs in CRC. Among them, lnc273–31 and lnc273–34 are seen to be depressed by p53 activity, which otherwise are responsible for tumor initiation, EMT, migration, invasion, chemoresistance, as well as CSC self-renewal. However, an establishment of such activities is observed due to the strong overexpression of these lncRNAs in CRC samples with a p53-R273 mutation [212]. An interesting study by Yue Gao et al. identified 5561 lncRNAs whose expression was affected by somatic mutations across 17 cancer types, with the highest (581) being in LUAD and the lowest (5) in colon adenocarcinoma. The majority of them were located in chromosomes 1 and 17. Around 61% were seen to have a downregulating expression. About 82% of these lncRNAs were affected solely by somatic mutations while the rest were observed to have a mixed impact of somatic mutations along with its effects on other factors, such as miRNA, gene, and transcription factor expression, methylation, etc. Several somatic mutations were observed to be linked with the transcription factor-binding sites [213].

LncRNASNP2 is a database with extensive information on several mutations and SNPs linked to mouse and human lncRNAs. Apart from mutational effects on the structure and functions of lncRNAs, it has comprehensive data on cancer-related mutational impacts, interactions with miRNAs, and lncRNA expression patterns across various diseases and 20 types of tumors [214]. The Lnc2Cancer database comprises about 1057 associations among 86 human tumors and 531 lncRNAs. It gives information on expression profiles of lncRNAs in respective cancer types along with the experimental details [215]. lncExplore is a database with extensive information on cancer-specific lncRNAs expression patterns and regulatory functions across 24 human tumors, genomic annotations, and potential biomarker candidates [216].

## 11. LncRNAs as Markers and Therapeutic Targets

On an optimistic note, lncRNAs showing cancer/tissue-specific expression can be utilized as biomarkers as well as aid in therapies targeting CSCs, and different antagonizing lncRNAs. In addition, addressing lncRNAs linked to certain tumor-associated mutations can also hold therapeutic potential. For instance, in liver tumors with ARID1A mutations, lncRNA MVIH can be targeted to decrease angiogenesis and cancer progression. In CRC, lnc273–31 and lnc273–34 can be employed as possible prognostic markers in the case of patients exhibiting the p53-R273H mutation. Cancer initiation and its developmental stages can be anticipated using SNPs associated with frequent tumor-related lncRNAs as biomarkers [217].

Radiation and chemotherapy are considered as the best options for treating cancer; however, lncRNAs, apart from their roles in tumor initiation and progression, are also accountable for imparting radiation and chemo-drug resistance. This poses a major obstacle in anti-tumor therapies, hence it is necessary to develop therapeutics targeting such lncRNAs such as lncARSR, lncRNA-cCSC1, and lncRNA THOR, for incurring chemo-drug resistance, and lncRNA TUG1 for causing radio-resistance, and many more that are responsible in different cancer types. From PCA3, the first long non-coding RNA approved by the FDA as a diagnostic marker for prostate cancer, till today, the field of lncRNAs as biomarkers is continuing to evolve.

In breast cancer, lncRNA HOTAIR is seen to maintain the stemness and migration properties of CSCs. It is also involved in cancer development and metastasis. Chen J et al. targeted the lncRNAs activity by Genistein and Calycosin. These drugs functioned by deactivating the PI3K/AKT signaling pathway, which further led to reduced HOTAIR expression [218]. In HCC, lncRNA ICR is observed to actively induce the expression of CD54 by creating an RNA duplex in order to effectively stabilize its mRNA activity. Thus, functioning in the maintenance of ICAM-1 positive LCSC self-renewal properties and the development of portal vein tumor thrombus. CD54 expression can be decreased by developing lncRNA ICR as a possible target for HCC therapy [219]. A four lncRNA signature was developed as a prognostic marker for GBM patients. Analyzing the changing expression profiles of the lncRNAs during tumor development and progression serves as an accurate diagnostic measure. This signature was observed to cluster with nine immune-associated networks, and four pathways [220]. These lncs may also be targeted for glioma therapeutics. LncUEGC1 can be utilized as a promising marker for the initial stages of gastric cancer. Its encapsulation in exosomes was seen to be significantly sensitive, with favorable stability [221]. In papillary thyroid carcinoma, lncRNA BANCR is responsible for maintaining stemness and tumor growth via the c-RAF/MEK/ERK pathway; additionally, it is observed to influence the expression of CSC markers LGR5 and EpCAM, this eventually leads to tumor development. Due to its specificity, it can serve as a therapeutic target to hinder cancer development [222].

In liver cancer, overexpression of lncRNA HAND2-AS1 is observed. It is responsible for activating the BMP signaling pathway by recruiting INO80 complex to bone-morphogenic protein R1A promoter; this results in the maintenance of CSC stemness and promotes tumor initiation and progression. Hence, it can be used as a diagnostic indicator as well as a therapeutic target [223]. In CRC, BCAR4 is observed to recruit miR-665 and induce downstream signal transducers activating the STAT3 pathway. Overexpression of the lncRNA is linked with the self-renewal activity of CSCs and the proliferation and spread of ALDH+ cells [224]. In breast cancer, LncCCAT1 is seen to be highly expressed; it associates with miR-204/211, and miR-148a/152, and interacts with ANXA2, to eventually positively influence TCF4 in such a way that the WNT signaling pathway is activated. This maintains CSC self-renewal capabilities along with high tumorigenic activities [225]. In liver cancer, lncRNA FAM99A was seen to act as a tumor suppressor, and its upregulation was seen to reduce colony formation, proliferation, and invasion of HCC cells making it a possible prognostic marker in HCC [226]. As lncRNA HDAC2 expression profiles are directly proportional to the severity of HCC, it may also act as a diagnostic marker during initial stages and a promising drug target during increased expression levels.

Depending on their expression profiles and cancer specificities, a number of lncRNAs have emerged as potential candidates as biomarkers as well as therapeutic targets. Bhan et al. have provided the detailed review on genome-wide association studies of tumor sample and provided a large number of lncRNAs associated with multiple types of cancer [227]. However, there is a need for GWAS analysis on cancer stem cells which may provide insights on novel lncRNA. Several hurdles still remain before they can be widely used; studies are required to obtain clarity on the interaction of lncRNAs with miRNAs, proteins, and regulatory elements that influence several hallmarks of cancer.

## 12. Conclusions

Over the years, it has become quite evident that non-coding RNAs no longer belong to the category of evolutionary junk. These RNAs have been observed to participate in numerous crucial cellular processes, and their alterations or mutations may lead to life-threatening effects. They are also observed to participate in various diseases and disorders, including cancer. This review was focused on the interaction and influences of lncRNAs on different tumor stem-cell signaling pathways. LncRNAs are accountable for having an oncogenic role or tumor-suppressive role in modulating a wide range of cancers. Similarly, their overexpression or decreased expression is responsible for either positively or negatively regulating the cancer stem-cell signaling pathways in a way such that they behave as oncogenic or tumor-suppressive agents. Various interactions and regulations of different lncRNAs have become known but more research is needed to fully understand the underlying mechanisms by which lncRNAs carry out such regulations. This will not only help in categorizing different lncRNAs as tumor-specific biomarkers but also open a new scope of therapeutics to target CSCs, such as the knockdown of lncRNAs or complexing them with miRNAs, hindering their regulation.

## Data Availability

Not applicable for this article.

## Abbreviations

	Description
ADAM	A disintegrin and metalloproteinase
Akt	Protein kinase B
ALDH	Aldehyde dehydrogenases
BCSC	Breast cancer stem cells
CADM1	Cell adhesion molecule1
CD	Cluster of differentiation
C-Myc	Cellular Myelocytomatosis transcriptional factor
CRNDE	Colorectal Neoplasia Differentially Expressed
CSF	Colony Stimulating Factor
CTNNB1	Catenin Beta 1
CUDR	Cancer upregulated drug resistant
EGF	Epidermal growth factor
ERK	Extracellular signal-regulated kinase
FGF	Fibroblast growth factor
FOXD2-AS1	FOXD2 Adjacent Opposite Strand RNA 1
GAS1	Growth arrest-specific protein 1
HGF	Hepatocyte growth factor
HK2	Hexokinase 2
HOTAIR	HOX antisense intergenic RNA
IFNGR	Interferon-gamma receptor
IFNK	Interferon-kappa
IL	Interleukin
KLF4	Krüppel-like factor 4
LGR	Leucine-rich repeat containing G-protein coupled receptor
MAPK	Mitogen-activated protein kinase
MRP1	Multidrug resistance associated protein
mTOR	Mammalian target of rapamycin
N1ICD	NOTCH1 intracellular domain
NESTIN	Neuroepithelial stem cell protein
NF-κB	Nuclear Factor kappaB
OCT	Octamer-binding transcription factor 4
PCGEM1	Prostate Gene Expression Marker 1
PDGF	Platelet-derived growth factor
PFKFB3	6-phosphofructo-2-kinase/fructose-2,6-biphosphatase 3
PI3K	Phosphatidylinositol 3-kinase
PNUTS1	Protein phosphatase 1 nuclear-targeting subunit
Prmt5	Protein arginine methyltransferase 5
PRNCR1	Prostate cancer-associated non-coding RNA1
PTEN	Phosphatase and Tensin Homolog deleted on Chromosome 10
RAF	Rapidly accelerated fibrosarcoma
SMAD	Suppressor of Mothers against Decapentaplegic
SNIP1	SMAD Nuclear Interacting Protein 1
STAT	Signal transducer and activator of transcription
TAZ	Transcriptional coactivator with a PDZ-binding domain
TCF/LEF	T-cell factor/lymphoid enhancer factor
TGF-β	Transforming growth factor-β
VEGF	Vascular endothelial growth factor
VEGFA	Vascular endothelial growth factor A
WNT	Wingless/Integrated
XIST	X-inactive specific transcript
YAP	Yes-associated protein

## References

[1] Palazzo A.F., Lee E.S. (2015). Non-Coding RNA: What Is Functional and What Is Junk?. Front. Genet..

[2] Borah A., Raveendran S., Rochani A., Maekawa T., Kumar D.S. (2015). Targeting Self-Renewal Pathways in Cancer Stem Cells: Clinical Implications for Cancer Therapy. Oncogenesis.

[3] Guzel E., Okyay T.M., Yalcinkaya B., Karacaoglu S., Gocmen M., Akcakuyu M.H. (2020). Tumor Suppressor and Oncogenic Role of Long Non-Coding RNAs in Cancer. North. Clin. Istanb..

[4] Zhang H., Chen Z., Wang X., Huang Z., He Z., Chen Y. (2013). Long Non-Coding RNA: A New Player in Cancer. J. Hematol. Oncol..

[5] Shahrouki P., Larsson E. (2012). The Non-Coding Oncogene: A Case of Missing DNA Evidence?. Front. Genet..

[6] Muller J. (1838). Uber Den Feineran Bau and Die For-Man Der Krankhauten Geschwulste.

[7] Cohnheim J. (1877). Vorlesungen Über Allgemeine Pathologie: Ein Handbuch für Aerzte Und Studirende.

[8] Lapidot T., Sirard C., Vormoor J., Murdoch B., Hoang T., Caceres-Cortes J., Minden M., Paterson B., Caligiuri M.A., Dick J.E. (1994). A Cell Initiating Human Acute Myeloid Leukaemia after Transplantation into SCID Mice. Nature.

[9] Bonnet D., Dick J.E. (1997). Human Acute Myeloid Leukemia Is Organized as a Hierarchy That Originates from a Primitive Hematopoietic Cell. Nat. Med..

[10] Singh S.K., Hawkins C., Clarke I.D., Squire J.A., Bayani J., Hide T., Henkelman R.M., Cusimano M.D., Dirks P.B. (2004). Identification of Human Brain Tumour Initiating Cells. Nature.

[11] Chiba T., Kita K., Zheng Y.-W., Yokosuka O., Saisho H., Iwama A., Nakauchi H., Taniguchi H. (2006). Side Population Purified from Hepatocellular Carcinoma Cells Harbors Cancer Stem Cell–like Properties. Hepatology.

[12] Yamashita T., Wang X.W. (2013). Cancer Stem Cells in the Development of Liver Cancer. J. Clin. Investig..

[13] Li C., Heidt D.G., Dalerba P., Burant C.F., Zhang L., Adsay V., Wicha M., Clarke M.F., Simeone D.M. (2007). Identification of Pancreatic Cancer Stem Cells. Cancer Res..

[14] Ricci-Vitiani L., Lombardi D.G., Pilozzi E., Biffoni M., Todaro M., Peschle C., De Maria R. (2007). Identification and Expansion of Human Colon-Cancer-Initiating Cells. Nature.

[15] Schatton T., Murphy G.F., Frank N.Y., Yamaura K., Waaga-Gasser A.M., Gasser M., Zhan Q., Jordan S., Duncan L.M., Weishaupt C. (2008). Identification of Cells Initiating Human Melanomas. Nature.

[16] Yang Y.M., Chang J.W. (2008). Bladder Cancer Initiating Cells (BCICs) Are among EMA-CD44v6+ Subset: Novel Methods for Isolating Undetermined Cancer Stem (Initiating) Cells. Cancer Investig..

[17] Takaishi S., Okumura T., Tu S., Wang S.S.W., Shibata W., Vigneshwaran R., Gordon S.A.K., Shimada Y., Wang T.C. (2009). Identification of Gastric Cancer Stem Cells Using the Cell Surface Marker CD44. Stem Cells.

[18] Alamgeer M., Peacock C.D., Matsui W., Ganju V., Watkins D.N. (2013). Cancer Stem Cells in Lung Cancer: Evidence and Controversies. Respirology.

[19] Han J., Fujisawa T., Husain S.R., Puri R.K. (2014). Identification and Characterization of Cancer Stem Cells in Human Head and Neck Squamous Cell Carcinoma. BMC Cancer.

[20] Baccelli I., Trumpp A. (2012). The Evolving Concept of Cancer and Metastasis Stem Cells. J. Cell Biol..

[21] Nejad A.E., Najafgholian S., Rostami A., Sistani A., Shojaeifar S., Esparvarinha M., Nedaeinia R., Javanmard S.H., Taherian M., Ahmadlou M. (2021). The Role of Hypoxia in the Tumor Microenvironment and Development of Cancer Stem Cell: A Novel Approach to Developing Treatment. Cancer Cell Int..

[22] Lizárraga-Verdugo E., Avendaño-Félix M., Bermúdez M., Ramos-Payán R., Pérez-Plasencia C., Aguilar-Medina M. (2020). Cancer Stem Cells and Its Role in Angiogenesis and Vasculogenic Mimicry in Gastrointestinal Cancers. Front. Oncol..

[23] Jones D.L., Wagers A.J. (2008). No Place like Home: Anatomy and Function of the Stem Cell Niche. Nat. Rev. Mol. Cell Biol..

[24] Plaks V., Kong N., Werb Z. (2015). The Cancer Stem Cell Niche: How Essential Is the Niche in Regulating Stemness of Tumor Cells?. Cell Stem Cell.

[25] Friedmann-Morvinski D., Verma I.M. (2014). Dedifferentiation and Reprogramming: Origins of Cancer Stem Cells. EMBO Rep..

[26] Takebe N., Miele L., Harris P.J., Jeong W., Bando H., Kahn M., Yang S.X., Ivy S.P. (2015). Targeting Notch, Hedgehog, and Wnt Pathways in Cancer Stem Cells: Clinical Update. Nat. Rev. Clin. Oncol..

[27] Chen K., Huang Y.H., Chen J.L. (2013). Understanding and Targeting Cancer Stem Cells: Therapeutic Implications and Challenges. Acta Pharmacol. Sin..

[28] Ajani J.A., Song S., Hochster H.S., Steinberg I.B. (2015). Cancer Stem Cells: The Promise and the Potential. Semin. Oncol..

[29] Oskarsson T., Batlle E., Massagué J. (2014). Metastatic Stem Cells: Sources, Niches, and Vital Pathways. Cell Stem Cell.

[30] Ucuzian A.A., Gassman A.A., East A.T., Greisler H.P. (2010). Molecular Mediators of Angiogenesis. J. Burn Care Res..

[31] Borggrefe T., Oswald F. (2009). The Notch Signaling Pathway: Transcriptional Regulation at Notch Target Genes. Cell. Mol. Life Sci..

[32] Kwon S.M., Alev C., Lee S.H., Asahara T. (2012). The Molecular Basis of Notch Signaling: A Brief Overview. Adv. Exp. Med. Biol..

[33] Liu X., Fan D. (2015). The Epithelial-Mesenchymal Transition and Cancer Stem Cells: Functional and Mechanistic Links. Curr. Pharm. Des..

[34] Zhu P., Wang Y., Du Y., He L., Huang G., Zhang G., Yan X., Fan Z. (2015). C8orf4 Negatively Regulates Self-Renewal of Liver Cancer Stem Cells via Suppression of NOTCH2 Signalling. Nat. Commun..

[35] Wu W.R., Zhang R., Shi X.D., Yi C., Xu L.B., Liu C. (2016). Notch2 Is a Crucial Regulator of Self-Renewal and Tumorigenicity in Human Hepatocellular Carcinoma Cells. Oncol. Rep..

[36] Roessler E., Belloni E., Gaudenz K., Jay P., Berta P., Scherer S.W., Tsui L.-C., Muenke M. (1996). Mutations in the Human Sonic Hedgehog Gene Cause Holoprosencephaly. Nat. Genet..

[37] Taipale J., Beachy P.A. (2001). The Hedgehog and Wnt Signalling Pathways in Cancer. Nature.

[38] Choudhry Z., Rikani A.A., Choudhry A.M., Tariq S., Zakaria F., Asghar M.W., Sarfraz M.K., Haider K., Shafiq A.A., Mobassarah N.J. (2014). Sonic Hedgehog Signalling Pathway: A Complex Network. Ann. Neurosci..

[39] Katoh Y., Katoh M. (2005). Hedgehog Signaling Pathway and Gastric Cancer. Cancer Biol. Ther..

[40] Campbell V., Copland M. (2015). Hedgehog Signaling in Cancer Stem Cells: A Focus on Hematological Cancers. Stem Cells Cloning Adv. Appl..

[41] Huang F.-T., Zhuan-Sun Y.-X., Zhuang Y.-Y., Wei S.-L., Tang J., Chen W.-B., Zhang S.-N. (2012). Inhibition of Hedgehog Signaling Depresses Self-Renewal of Pancreatic Cancer Stem Cells and Reverses Chemoresistance. Int. J. Oncol..

[42] Bhavanasi D., Klein P.S. (2016). Wnt Signaling in Normal and Malignant Stem Cells. Curr. Stem Cell Rep..

[43] Nusse R. (2005). Wnt Signaling in Disease and in Development. Cell Res..

[44] Polakis P. (2012). Wnt Signaling in Cancer. Cold Spring Harb. Perspect. Biol..

[45] Baron R., Kneissel M. (2013). WNT Signaling in Bone Homeostasis and Disease: From Human Mutations to Treatments. Nat. Med..

[46] MacDonald B.T., Tamai K., He X. (2009). Wnt/β-Catenin Signaling: Components, Mechanisms, and Diseases. Dev. Cell.

[47] Lin C.H., Ji T., Chen C.-F., Hoang B.H. (2014). Wnt Signaling in Osteosarcoma. Adv. Exp. Med. Biol..

[48] Prosperi J.R., Goss K.H. (2010). A Wnt-Ow of Opportunity: Targeting the Wnt/b-Catenin Pathway in Breast Cancer. Curr. Drug Targets.

[49] Ashihara E., Takada T., Maekawa T. (2015). Targeting the Canonical Wnt/β-Catenin Pathway in Hematological Malignancies. Cancer Sci..

[50] Majeti R., Becker M.W., Tian Q., Lee T.L.M., Yan X., Liu R., Chiang J.H., Hood L., Clarke M.F., Weissman I.L. (2009). Dysregulated Gene Expression Networks in Human Acute Myelogenous Leukemia Stem Cells. Proc. Natl. Acad. Sci. USA.

[51] Malanchi I., Peinado H., Kassen D., Hussenet T., Metzger D., Chambon P., Huber M., Hohl D., Cano A., Birchmeier W. (2008). Cutaneous Cancer Stem Cell Maintenance Is Dependent on β-Catenin Signalling. Nature.

[52] Peifer M., Polakis P. (2000). Wnt Signaling in Oncogenesis and Embryogenesis—A Look Outside the Nucleus. Science.

[53] Ivashkiv L.B. (2000). Jak-STAT Signaling Pathways in Cells of the Immune System. Rev. Immunogenet..

[54] Schuringa J.J., Chung K.Y., Morrone G., Moore M.A.S. (2004). Constitutive Activation of STAT5A Promotes Human Hematopoietic Stem Cell Self-Renewal and Erythroid Differentiation. J. Exp. Med..

[55] Yu H., Lee H., Herrmann A., Buettner R., Jove R. (2014). Revisiting STAT3 Signalling in Cancer: New and Unexpected Biological Functions. Nat. Rev. Cancer.

[56] Leonard W.J., O’Shea J.J. (1998). JAKS AND STATS: Biological Implications. Annu. Rev. Immunol..

[57] Zhang H., Wang Z.Z. (2008). Mechanisms That Mediate Stem Cell Self-Renewal and Differentiation. J. Cell. Biochem..

[58] Schroeder A., Herrmann A., Cherryholmes G., Kowolik C., Buettner R., Pal S., Yu H., Müller-Newen G., Jove R. (2014). Loss of Androgen Receptor Expression Promotes a Stem-like Cell Phenotype in Prostate Cancer through STAT3 Signaling. Cancer Res..

[59] Hernandez-Vargas H., Ouzounova M., le Calvez-Kelm F., Lambert M.P., McKay-Chopin S., Tavtigian S.V., Puisieux A., Matar C., Herceg Z. (2011). Methylome Analysis Reveals Jak-STAT Pathway Deregulation in Putative Breast Cancer Stem Cells. Epigenetics.

[60] Sakaki-Yumoto M., Katsuno Y., Derynck R. (2013). TGF-β Family Signaling in Stem Cells. Biochim. Et Biophys. Acta (BBA)-Gen. Subj..

[61] Miyazono K. (2000). TGF-β Signaling by Smad Proteins. Cytokine Growth Factor Rev..

[62] Shi Y., Massagué J. (2003). Mechanisms of TGF-β Signaling from Cell Membrane to the Nucleus. Cell.

[63] Heldin C.H., Miyazono K., Dijke P.T. (1997). TGF-β Signalling from Cell Membrane to Nucleus through SMAD Proteins. Nature.

[64] Jiang F., Mu J., Wang X., Ye X., Si L., Ning S., Li Z., Li Y. (2014). The Repressive Effect of MiR-148a on TGF Beta-SMADs Signal Pathway Is Involved in the Glabridin-Induced Inhibition of the Cancer Stem Cells-like Properties in Hepatocellular Carcinoma Cells. PLoS ONE.

[65] Xia Y., Shen S., Verma I.M. (2014). NF-ΚB, an Active Player in Human Cancers. Cancer Immunol. Res..

[66] Hoesel B., Schmid J.A. (2013). The Complexity of NF-ΚB Signaling in Inflammation and Cancer. Mol. Cancer.

[67] Kaltschmidt B., Greiner J.F.W., Kadhim H.M., Kaltschmidt C. (2018). Subunit-Specific Role of NF-ΚB in Cancer. Biomedicines.

[68] Riedlinger T., Haas J., Busch J., Van de Sluis B., Kracht M., Schmitz M.L. (2018). The Direct and Indirect Roles of NF-ΚB in Cancer: Lessons from Oncogenic Fusion Proteins and Knock-in Mice. Biomedicines.

[69] Baud V., Collares D. (2016). Post-Translational Modifications of RelB NF-ΚB Subunit and Associated Functions. Cells.

[70] Hanahan D., Weinberg R.A. (2011). Hallmarks of Cancer: The Next Generation. Cell.

[71] Taniguchi K., Karin M. (2018). NF-ΚB, Inflammation, Immunity and Cancer: Coming of Age. Nat. Rev. Immunol..

[72] Terzić J., Grivennikov S., Karin E., Karin M. (2010). Inflammation and Colon Cancer. Gastroenterology.

[73] Lawrence T., Fong C. (2010). The Resolution of Inflammation: Anti-Inflammatory Roles for NF-ΚB. Int. J. Biochem. Cell Biol..

[74] Saccani A., Schioppa T., Porta C., Biswas S.K., Nebuloni M., Vago L., Bottazzi B., Colombo M.P., Mantovani A., Sica A. (2006). P50 Nuclear Factor-ΚB Overexpression in Tumor-Associated Macrophages Inhibits M1 Inflammatory Responses and Antitumor Resistance. Cancer Res..

[75] Li M., Li J., Ding X., He M., Cheng S.-Y. (2010). MicroRNA and Cancer. AAPS J..

[76] Lu J., Getz G., Miska E.A., Alvarez-Saavedra E., Lamb J., Peck D., Sweet-Cordero A., Ebert B.L., Mak R.H., Ferrando A.A. (2005). MicroRNA Expression Profiles Classify Human Cancers. Nature.

[77] Cheng J., Guo J.M., Xiao B.X., Miao Y., Jiang Z., Zhou H., Li Q.N. (2011). PiRNA, the New Non-Coding RNA, Is Aberrantly Expressed in Human Cancer Cells. Clin. Chim. Acta.

[78] Romano G., Veneziano D., Acunzo M., Croce C.M. (2017). Small Non-Coding RNA and Cancer. Carcinogenesis.

[79] Wang K.C., Chang H.Y. (2011). Molecular Mechanisms of Long Noncoding RNAs. Mol. Cell.

[80] Li W., Jiang P., Sun X., Xu S., Ma X., Zhan R. (2016). Suppressing H19 Modulates Tumorigenicity and Stemness in U251 and U87MG Glioma Cells. Cell. Mol. Neurobiol..

[81] Peng F., Li T.T., Wang K.L., Xiao G.Q., Wang J.H., Zhao H.D., Kang Z.J., Fan W.J., Zhu L.L., Li M. (2017). H19/Let-7/LIN28 Reciprocal Negative Regulatory Circuit Promotes Breast Cancer Stem Cell Maintenance. Cell Death Dis..

[82] Feng S., Yao J., Chen Y., Geng P., Zhang H., Ma X., Zhao J., Yu X. (2015). Expression and Functional Role of Reprogramming-Related Long Noncoding RNA (LincRNA-ROR) in Glioma. J. Mol. Neurosci..

[83] Wu M., An J., Zheng Q., Xin X., Lin Z., Li X., Li H., Lu D. (2016). Double Mutant P53 (N340Q/L344R) Promotes Hepatocarcinogenesis through Upregulation of Pim1 Mediated by PKM2 and LncRNA CUDR. Oncotarget.

[84] Pu H., Zheng Q., Li H., Wu M., An J., Gui X., Li T., Lu D. (2015). CUDR Promotes Liver Cancer Stem Cell Growth through Upregulating TERT and C-Myc. Oncotarget.

[85] Li L., Dang Q., Xie H., Yang Z., He D., Liang L., Song W., Yeh S., Chang C. (2016). Correction: Infiltrating Mast Cells Enhance Prostate Cancer Invasion via Altering LncRNA-HOTAIR/PRC2-Androgen Receptor (AR)-MMP9 Signals and Increased Stem/Progenitor Cell Population. Oncotarget.

[86] Alves C.P., Fonseca A.S., Muys B.R., Bueno R.D.B.E.L., Burger M.C., De Souza J.E.S., Valente V., Zago M.A., Silva W.A. (2013). Brief Report: The LincRNA Hotair Is Required for Epithelial-to-Mesenchymal Transition and Stemness Maintenance of Cancer Cell Lines. Stem Cells.

[87] Galasso M., Dama P., Previati M., Sandhu S., Palatini J., Coppola V., Warner S., Sana M.E., Zanella R., Abujarour R. (2014). A Large Scale Expression Study Associates Uc.283-plus LncRNA with Pluripotent Stem Cells and Human Glioma. Genome Med..

[88] Wang Y., Wang Y., Li J., Zhang Y., Yin H., Han B. (2015). CRNDE, a Long-Noncoding RNA, Promotes Glioma Cell Growth and Invasion through MTOR Signaling. Cancer Lett..

[89] Ghafouri-Fard S., Dashti S., Farsi M., Taheri M., Mousavinejad S.A. (2021). X-Inactive-Specific Transcript: Review of Its Functions in the Carcinogenesis. Front. Cell Dev. Biol..

[90] Yang L., Lin C., Jin C., Yang J.C., Tanasa B., Li W., Merkurjev D., Ohgi K.A., Meng D., Zhang J. (2013). LncRNA-Dependent Mechanisms of Androgen-Receptor-Regulated Gene Activation Programs. Nature.

[91] Popadiuk C.M., Xiong J., Wells M.G., Andrews P.G., Dankwa K., Hirasawa K., Lake B.B., Kao K.R. (2006). Antisense Suppression of Pygopus2 Results in Growth Arrest of Epithelial Ovarian Cancer. Clin. Cancer Res..

[92] Zhou Y., Zhong Y., Wang Y., Zhang X., Batista D.L., Gejman R., Ansell P.J., Zhao J., Weng C., Klibanski A. (2007). Activation of P53 by MEG3 Non-Coding RNA. J. Biol. Chem..

[93] Gibb E.A., Brown C.J., Lam W.L. (2011). The Functional Role of Long Non-Coding RNA in Human Carcinomas. Mol. Cancer.

[94] Zhang X., Gejman R., Mahta A., Zhong Y., Rice K.A., Zhou Y., Cheunsuchon P., Louis D.N., Klibanski A. (2010). Maternally Expressed Gene 3, an Imprinted Noncoding RNA Gene, Is Associated with Meningioma Pathogenesis and Progression. Cancer Res..

[95] Benetatos L., Hatzimichael E., Dasoula A., Dranitsaris G., Tsiara S., Syrrou M., Georgiou I., Bourantas K.L. (2010). CpG Methylation Analysis of the MEG3 and SNRPN Imprinted Genes in Acute Myeloid Leukemia and Myelodysplastic Syndromes. Leuk. Res..

[96] Braconi C., Kogure T., Valeri N., Huang N., Nuovo G., Costinean S., Negrini M., Miotto E., Croce C.M., Patel T. (2011). MicroRNA-29 Can Regulate Expression of the Long Non-Coding RNA Gene MEG3 in Hepatocellular Cancer. Oncogene.

[97] Chiba S. (2006). Concise Review: Notch Signaling in Stem Cell Systems. Stem Cells.

[98] Karamboulas C., Ailles L. (2013). Developmental Signaling Pathways in Cancer Stem Cells of Solid Tumors. Biochim. Biophys. Acta (BBA)-Gen. Subj..

[99] Ellisen L.W., Bird J., West D.C., Soreng A.L., Reynolds T.C., Smith S.D., Sklar J. (1991). TAN-1, the Human Homolog of the Drosophila Notch Gene, Is Broken by Chromosomal Translocations in T Lymphoblastic Neoplasms. Cell.

[100] Ma Y.-C., Shi C., Zhang Y.-N., Wang L.-G., Liu H., Jia H.-T., Zhang Y.-X., Sarkar F.H., Wang Z.-S. (2012). The Tyrosine Kinase C-Src Directly Mediates Growth Factor-Induced Notch-1 and Furin Interaction and Notch-1 Activation in Pancreatic Cancer Cells. PLoS ONE.

[101] Wu F., Stutzman A., Mo Y.Y. (2007). Notch Signaling and Its Role in Breast Cancer. Front. Biosci..

[102] Song L.L., Peng Y., Yun J., Rizzo P., Chaturvedi V., Weijzen S., Kast W.M., Stone P.J.B., Santos L., Loredo A. (2008). Notch-1 Associates with IKKα and Regulates IKK Activity in Cervical Cancer Cells. Oncogene.

[103] Qiao L., Wong B.C.Y. (2009). Role of Notch Signaling in Colorectal Cancer. Carcinogenesis.

[104] Zhou W., Fu X.-Q., Zhang L.-L., Zhang J., Huang X., Lu X.-H., Shen L., Liu B.-N., Liu J., Luo H.-S. (2013). The AKT1/NF-KappaB/Notch1/PTEN Axis Has an Important Role in Chemoresistance of Gastric Cancer Cells. Cell Death Dis..

[105] Zhang Y., Li B., Ji Z.Z., Zheng P.S. (2010). Notch1 Regulates the Growth of Human Colon Cancers. Cancer.

[106] Ranganathan P., Weaver K.L., Capobianco A.J. (2011). Notch Signalling in Solid Tumours: A Little Bit of Everything but Not All the Time. Nat. Rev. Cancer.

[107] Abel E.V., Kim E.J., Wu J., Hynes M., Bednar F., Proctor E., Wang L., Dziubinski M.L., Simeone D.M. (2014). The Notch Pathway Is Important in Maintaining the Cancer Stem Cell Population in Pancreatic Cancer. PLoS ONE.

[108] Kannan S., Sutphin R.M., Hall M.G., Golfman L.S., Fang W., Nolo R.M., Akers L.J., Hammitt R.A., McMurray J.S., Kornblau S.M. (2013). Notch Activation Inhibits AML Growth and Survival: A Potential Therapeutic Approach. J. Exp. Med..

[109] Lefort K., Mandinova A., Ostano P., Kolev V., Calpini V., Kolfschoten I., Devgan V., Lieb J., Raffoul W., Hohl D. (2007). Notch1 Is a P53 Target Gene Involved in Human Keratinocyte Tumor Suppression through Negative Regulation of ROCK1/2 and MRCKα Kinases. Genes Dev..

[110] Konishi J., Yi F., Chen X., Vo H., Carbone D.P., Dang T.P. (2009). Notch3 Cooperates with the EGFR Pathway to Modulate Apoptosis through the Induction of Bim. Oncogene.

[111] Viatour P., Ehmer U., Saddic L.A., Dorrell C., Andersen J.B., Lin C., Zmoos A.F., Mazur P.K., Schaffer B.E., Ostermeier A. (2011). Notch Signaling Inhibits Hepatocellular Carcinoma Following Inactivation of the RB Pathway. J. Exp. Med..

[112] Gupta A., Wang Y., Browne C., Kim S., Case T., Paul M., Wills M.L., Matusik R.J. (2008). Neuroendocrine Differentiation in the 12T-10 Transgenic Prostate Mouse Model Mimics Endocrine Differentiation of Pancreatic Beta Cells. Prostate.

[113] Parr C., Watkins G., Jiang W.G. (2004). The Possible Correlation of Notch-1 and Notch-2 with Clinical Outcome and Tumour Clinicopathological Parameters in Human Breast Cancer. Int. J. Mol. Med..

[114] Li Y., Huo J., He J., Ma X. (2021). LncRNA MONC Suppresses the Malignant Phenotype of Endometrial Cancer Stem Cells and Endometrial Carcinoma Cells by Regulating the MiR-636/GLCE Axis. Cancer Cell Int..

[115] Tan J., Qiu K., Li M., Liang Y. (2015). Double-Negative Feedback Loop between Long Non-Coding RNA TUG1 and MiR-145 Promotes Epithelial to Mesenchymal Transition and Radioresistance in Human Bladder Cancer Cells. FEBS Lett..

[116] Cao W.J., Wu H.L., He B.S., Zhang Y.S., Zhang Z.Y. (2013). Analysis of Long Non-Coding RNA Expression Profiles in Gastric Cancer. World J. Gastroenterol..

[117] Zhang Q., Geng P.L., Yin P., Wang X.L., Jia J.P., Yao J. (2013). Down-Regulation of Long Non-Coding RNA TUG1 Inhibits Osteosarcoma Cell Proliferation and Promotes Apoptosis. Asian Pac. J. Cancer Prev..

[118] Zhang E.-B., Yin D.-D., Sun M., Kong R., Liu X.-H., You L.-H., Han L., Xia R., Wang K.-M., Yang J.-S. (2014). P53-Regulated Long Non-Coding RNA TUG1 Affects Cell Proliferation in Human Non-Small Cell Lung Cancer, Partly through Epigenetically Regulating HOXB7 Expression. Cell Death Dis..

[119] Zhang M., Han Y., Zheng Y., Zhang Y., Zhao X., Gao Z., Liu X. (2020). ZEB1-Activated LINC01123 Accelerates the Malignancy in Lung Adenocarcinoma through NOTCH Signaling Pathway. Cell Death Dis..

[120] Huang R., Nie W., Yao K., Chou J. (2019). Depletion of the LncRNA RP11-567G11.1 Inhibits Pancreatic Cancer Progression. Biomed. Pharmacother..

[121] Huang G., Wang M., Li X., Wu J., Chen S., Du N., Li K., Wang J., Xu C., Ren H. (2019). TUSC7 Suppression of Notch Activation through Sponging MiR-146 Recapitulated the Asymmetric Cell Division in Lung Adenocarcinoma Stem Cells. Life Sci..

[122] Acebron S.P., Karaulanov E., Berger B.S., Huang Y.L., Niehrs C. (2014). Mitotic Wnt Signaling Promotes Protein Stabilization and Regulates Cell Size. Mol. Cell.

[123] Atlasi Y., Noori R., Gaspar C., Franken P., Sacchetti A., Rafati H., Mahmoudi T., Decraene C., Calin G.A., Merrill B.J. (2013). Wnt Signaling Regulates the Lineage Differentiation Potential of Mouse Embryonic Stem Cells through Tcf3 Down-Regulation. PLoS Genet..

[124] Clevers H., Loh K.M., Nusse R. (2014). An Integral Program for Tissue Renewal and Regeneration: Wnt Signaling and Stem Cell Control. Science.

[125] Green J.L., Inoue T., Sternberg P.W. (2008). Opposing Wnt Pathways Orient Cell Polarity during Organogenesis. Cell.

[126] Zhan T., Rindtorff N., Boutros M. (2017). Wnt Signaling in Cancer. Oncogene.

[127] Grumolato L., Liu G., Mong P., Mudbhary R., Biswas R., Arroyave R., Vijayakumar S., Economides A.N., Aaronson S.A. (2010). Canonical and Noncanonical Wnts Use a Common Mechanism to Activate Completely Unrelated Coreceptors. Genes Dev..

[128] Katoh M. (2017). Canonical and Non-Canonical WNT Signaling in Cancer Stem Cells and Their Niches: Cellular Heterogeneity, Omics Reprogramming, Targeted Therapy and Tumor Plasticity (Review). Int. J. Oncol..

[129] Dieter S.M., Glimm H., Ball C.R. (2017). Colorectal Cancer-initiating Cells Caught in the Act. EMBO Mol. Med..

[130] Kahn M. (2014). Can We Safely Target the WNT Pathway?. Nat. Rev. Drug Discov..

[131] Mirabelli C.K., Nusse R., Tuveson D.A., Williams B.O. (2019). Perspectives on the Role of Wnt Biology in Cancer. Sci. Signal..

[132] Amin N., Cavallaro U. (2012). The Wnt Signaling Pathways and Cell Adhesion. Front. Biosci..

[133] Schatoff E.M., Leach B.I., Dow L.E. (2017). WNT Signaling and Colorectal Cancer. Curr. Color. Cancer Rep..

[134] Klarmann G.J., Decker A., Farrar W.L. (2008). Epigenetic Gene Silencing in the Wnt Pathway in Breast Cancer. Epigenetics.

[135] Wang Y., He L., Du Y., Zhu P., Huang G., Luo J., Yan X., Ye B., Li C., Xia P. (2015). The Long Noncoding RNA LncTCF7 Promotes Self-Renewal of Human Liver Cancer Stem Cells through Activation of Wnt Signaling. Cell Stem Cell.

[136] Todaro M., Gaggianesi M., Catalano V., Benfante A., Iovino F., Biffoni M., Apuzzo T., Sperduti I., Volpe S., Cocorullo G. (2014). CD44v6 Is a Marker of Constitutive and Reprogrammed Cancer Stem Cells Driving Colon Cancer Metastasis. Cell Stem Cell.

[137] Malanchi I., Santamaria-Martínez A., Susanto E., Peng H., Lehr H.A., Delaloye J.F., Huelsken J. (2012). Interactions between Cancer Stem Cells and Their Niche Govern Metastatic Colonization. Nature.

[138] Zhu P., Wu J., Wang Y., Zhu X., Lu T., Liu B., He L., Ye B., Wang S., Meng S. (2018). LncGata6 Maintains Stemness of Intestinal Stem Cells and Promotes Intestinal Tumorigenesis. Nat. Cell Biol..

[139] Li Z., Zhao L., Wang Q. (2016). Overexpression of Long Non-Coding RNA HOTTIP Increases Chemoresistance of Osteosarcoma Cell by Activating the Wnt/β-Catenin Pathway. Am. J. Transl. Res..

[140] Gui X., Li H., Li T., Pu H., Lu D. (2015). Long Noncoding RNA CUDR Regulates HULC and β-Catenin to Govern Human Liver Stem Cell Malignant Differentiation. Mol. Ther..

[141] Wang J., Lei Z.J., Guo Y., Wang T., Qin Z.Y., Xiao H.L., Fan L.L., Chen D.F., Bian X.W., Liu J. (2015). MiRNA-Regulated Delivery of LincRNA-P21 Suppresses β-Catenin Signaling and Tumorigenicity of Colorectal Cancer Stem Cells. Oncotarget.

[142] Luo M., Li Z., Wang W., Zeng Y., Liu Z., Qiu J. (2013). Long Non-Coding RNA H19 Increases Bladder Cancer Metastasis by Associating with EZH2 and Inhibiting E-Cadherin Expression. Cancer Lett..

[143] Zhu P., Wang Y., Huang G., Ye B., Liu B., Wu J., Du Y., He L., Fan Z. (2016). Lnc-β-Catm elicits EZH2-dependent β-catenin stabilization and sustains liver CSC self-renewal. Nat. Struct. Mol. Biol..

[144] Yuan S.-X., Wang J., Yang F., Tao Q.-F., Zhang J., Wang L.-L., Yang Y., Liu H., Wang Z.-G., Xu Q.-G. (2016). Long Noncoding RNA DANCR Increases Stemness Features of Hepatocellular Carcinoma by Derepression of CTNNB1. Hepatology.

[145] Yan J., Dang Y., Liu S., Zhang Y., Zhang G. (2016). LncRNA HOTAIR Promotes Cisplatin Resistance in Gastric Cancer by Targeting MiR-126 to Activate the PI3K/AKT/MRP1 Genes. Tumor Biol..

[146] Gatta L.B., Melocchi L., Bugatti M., Missale F., Lonardi S., Zanetti B., Cristinelli L., Belotti S., Simeone C., Ronca R. (2019). Hyper-Activation of STAT3 Sustains Progression of Non-Papillary Basal-Type Bladder Cancer via FOSL1 Regulome. Cancers.

[147] Kim J.H., Choi H.S., Kim S.L., Lee D.S. (2019). The PAK1-Stat3 Signaling Pathway Activates IL-6 Gene Transcription and Human Breast Cancer Stem Cell Formation. Cancers.

[148] White C.L., Jayasekara W.S.N., Picard D., Chen J., Watkins D.N., Cain J.E., Remke M., Gough D.J. (2019). A Sexually Dimorphic Role for STAT3 in Sonic Hedgehog Medulloblastoma. Cancers.

[149] Yun J.W., Lee S., Kim H.M., Chun S., Engleman E.G., Kim H.C., Kang E.S. (2019). A Novel Type of Blood Biomarker: Distinct Changes of Cytokine-Induced Stat Phosphorylation in Blood t Cells between Colorectal Cancer Patients and Healthy Individuals. Cancers.

[150] Severin F., Frezzato F., Visentin A., Martini V., Trimarco V., Carraro S., Tibaldi E., Maria Brunati A., Piazza F., Semenzato G. (2019). In Chronic Lymphocytic Leukemia the JAK2/STAT3 Pathway Is Constitutively Activated and Its Inhibition Leads to CLL Cell Death Unaffected by the Protective Bone Marrow Microenvironment. Cancers.

[151] Morgan E.L., Macdonald A. (2019). JAK2 Inhibition Impairs Proliferation and Sensitises Cervical Cancer Cells to Cisplatin-Induced Cell Death. Cancers.

[152] Basu R., Kulkarni P., Qian Y., Walsh C., Arora P., Davis E., Duran-Ortiz S., Funk K., Ibarra D., Kruse C. (2019). Growth Hormone Upregulates Melanocyte-Inducing Transcription Factor Expression and Activity via JAK2-STAT5 and SRC Signaling in GH Receptor-Positive Human Melanoma. Cancers.

[153] Maurer B., Kollmann S., Pickem J., Hoelbl-Kovacic A., Sexl V. (2019). STAT5A and STAT5B—Twins with Different Personalities in Hematopoiesis and Leukemia. Cancers.

[154] Moll H.P., Mohrherr J., Blaas L., Musteanu M., Stiedl P., Grabner B., Zboray K., König M., Stoiber D., Rülicke T. (2019). A Mouse Model to Assess STAT3 and STAT5A/B Combined Inhibition in Health and Disease Conditions. Cancers.

[155] Valle-Mendiola A., Soto-Cruz I. (2020). Energy Metabolism in Cancer: The Roles of STAT3 and STAT5 in the Regulation of Metabolism-Related Genes. Cancers.

[156] Seif F., Khoshmirsafa M., Aazami H., Mohsenzadegan M., Sedighi G., Bahar M. (2017). The Role of JAK-STAT Signaling Pathway and Its Regulators in the Fate of T Helper Cells. Cell Commun. Signal..

[157] Birnie R., Bryce S.D., Roome C., Dussupt V., Droop A., Lang S.H., Berry P.A., Hyde C.F., Lewis J.L., Stower M.J. (2008). Gene Expression Profiling of Human Prostate Cancer Stem Cells Reveals a Pro-Inflammatory Phenotype and the Importance of Extracellular Matrix Interactions. Genome Biol..

[158] Zhou J., Wulfkuhle J., Zhang H., Gu P., Yang Y., Deng J., Margolick J.B., Liotta L.A., Petricoin E., Zhang Y. (2007). Activation of the PTEN/MTOR/STAT3 Pathway in Breast Cancer Stem-like Cells Is Required for Viability and Maintenance. Proc. Natl. Acad. Sci. USA.

[159] Sherry M.M., Reeves A., Wu J.K., Cochran B.H. (2009). STAT3 Is Required for Proliferation and Maintenance of Multipotency in Glioblastoma Stem Cells. Stem Cells.

[160] Cook A.M., Li L., Ho Y., Lin A., Li L., Stein A., Forman S., Perrotti D., Jove R., Bhatia R. (2014). Role of Altered Growth Factor Receptor-Mediated JAK2 Signaling in Growth and Maintenance of Human Acute Myeloid Leukemia Stem Cells. Blood.

[161] Zhang H.F., Lai R. (2014). STAT3 in Cancer-Friend or Foe?. Cancers.

[162] Vultur A., Cao J., Arulanandam R., Turkson J., Jove R., Greer P., Craig A., Elliott B., Raptis L. (2004). Cell-to-Cell Adhesion Modulates Stat3 Activity in Normal and Breast Carcinoma Cells. Oncogene.

[163] Steinman R.A., Wentzel A., Lu Y., Stehle C., Grandis J.R. (2003). Activation of Stat3 by Cell Confluence Reveals Negative Regulation of Stat3 by Cdk2. Oncogene.

[164] Gkouveris I., Nikitakis N., Karanikou M., Rassidakis G., Sklavounou A. (2014). Erk1/2 Activation and Modulation of STAT3 Signaling in Oral Cancer. Oncol. Rep..

[165] Yan S., Li Z., Thiele C.J., Yan S., Li Z., Thiele C.J. (2013). Inhibition of STAT3 with Orally Active JAK Inhibitor, AZD1480, Decreases Tumor Growth in Neuroblastoma and Pediatric Sarcomas In Vitro and In Vivo. Oncotarget.

[166] Ivanov V.N., Bhoumik A., Krasilnikov M., Raz R., Owen-Schaub L.B., Levy D., Horvath C.M., Ronai Z. (2001). Cooperation between STAT3 and C-Jun Suppresses Fas Transcription. Mol. Cell.

[167] Barré B., Avril S., Coqueret O. (2003). Opposite Regulation of Myc and P21 Waf1 Transcription by STAT3 Proteins. J. Biol. Chem..

[168] Kroon P., Berry P.A., Stower M.J., Rodrigues G., Mann V.M., Simms M., Bhasin D., Chettiar S., Li C., Li P.K. (2013). JAK-STAT Blockade Inhibits Tumor Initiation and Clonogenic Recovery of Prostate Cancer Stem-like Cells. Cancer Res..

[169] Qu Y., Oyan A.M., Liu R., Hua Y., Zhang J., Hovland R., Popa M., Liu X., Brokstad K.A., Simon R. (2013). Generation of Prostate Tumor–Initiating Cells Is Associated with Elevation of Reactive Oxygen Species and IL-6/STAT3 Signaling. Cancer Res..

[170] Rybak A.P., Bristow R.G., Kapoor A. (2015). Prostate Cancer Stem Cells: Deciphering the Origins and Pathways Involved in Prostate Tumorigenesis and Aggression. Oncotarget.

[171] Zhao D., Pan C., Sun J., Gilbert C., Drews-Elger K., Azzam D.J., Picon-Ruiz M., Kim M., Ullmer W., El-Ashry D. (2014). VEGF Drives Cancer-Initiating Stem Cells through VEGFR-2/Stat3 Signaling to Upregulate Myc and SOX2. Oncogene.

[172] Gu L.Q., Xing X.L., Cai H., Si A.F., Hu X.R., Ma Q.Y., Zheng M.L., Wang R.Y., Li H.Y., Zhang X.P. (2018). Long Non-Coding RNA DILC Suppresses Cell Proliferation and Metastasis in Colorectal Cancer. Gene.

[173] Wang X., Sun W., Shen W., Xia M., Chen C., Xiang D., Ning B., Cui X., Li H., Li X. (2016). Long Non-Coding RNA DILC Regulates Liver Cancer Stem Cells via IL-6/STAT3 Axis. J. Hepatol..

[174] Iliopoulos D., Hirsch H.A., Struhl K. (2009). An Epigenetic Switch Involving NF-ΚB, Lin28, Let-7 MicroRNA, and IL6 Links Inflammation to Cell Transformation. Cell.

[175] Kagoya Y., Yoshimi A., Kataoka K., Nakagawa M., Kumano K., Arai S., Kobayashi H., Saito T., Iwakura Y., Kurokawa M. (2014). Positive Feedback between NF-ΚB and TNF-α Promotes Leukemia-Initiating Cell Capacity. J. Clin. Investig..

[176] Magagula L., Gagliardi M., Naidoo J., Mhlanga M. (2017). Lnc-Ing Inflammation to Disease. Biochem. Soc. Trans..

[177] Chen Z., Huang L., Wu Y., Zhai W., Zhu P., Gao Y. (2016). LncSOX4 Promotes the Self-Renewal of Liver Tumour-Initiating Cells through Stat3-Mediated SOX4 Expression. Nat. Commun..

[178] Li W., Chen Y., Nie X. (2020). Regulatory Mechanisms of LncRNAs and Their Target Gene Signaling Pathways in Laryngeal Squamous Cell Carcinoma. Front. Pharmacol..

[179] Yang C., Cai W.C., Dong Z.T., Guo J.W., Zhao Y.J., Sui C.J., Yang J. (2019). mei LncARSR Promotes Liver Cancer Stem Cells Expansion via STAT3 Pathway. Gene.

[180] Wu D.M., Zheng Z.H., Zhang Y.B., Fan S.H., Zhang Z.F., Wang Y.J., Zheng Y.L., Lu J. (2019). Down-Regulated LncRNA DLX6-AS1 Inhibits Tumorigenesis through STAT3 Signaling Pathway by Suppressing CADM1 Promoter Methylation in Liver Cancer Stem Cells. J. Exp. Clin. Cancer Res..

[181] Yang L., Xie G., Fan Q., Xie J. (2010). Activation of the Hedgehog-Signaling Pathway in Human Cancer and the Clinical Implications. Oncogene.

[182] Peacock C.D., Wang Q., Gesell G.S., Corcoran-Schwartz I.M., Jones E., Kim J., Devereux W.L., Rhodes J.T., Huff C.A., Beachy P.A. (2007). Hedgehog Signaling Maintains a Tumor Stem Cell Compartment in Multiple Myeloma. Proc. Natl. Acad. Sci. USA.

[183] Dembinski J.L., Krauss S. (2009). Characterization and Functional Analysis of a Slow Cycling Stem Cell-like Subpopulation in Pancreas Adenocarcinoma. Clin. Exp. Metastasis.

[184] Liu S., Dontu G., Mantle I.D., Patel S., Ahn N.S., Jackson K.W., Suri P., Wicha M.S. (2006). Hedgehog Signaling and Bmi-1 Regulate Self-Renewal of Normal and Malignant Human Mammary Stem Cells. Cancer Res..

[185] Long B., Zhu H., Zhu C., Liu T., Meng W. (2011). Activation of the Hedgehog Pathway in Chronic Myelogeneous Leukemia Patients. J. Exp. Clin. Cancer Res..

[186] Johnson R.L., Rothman A.L., Xie J., Goodrich L.V., Bare J.W., Bonifas J.M., Quinn A.G., Myers R.M., Cox D.R., Epstein E.H. (1996). Human Homolog of Patched, a Candidate Gene for the Basal Cell Nevus Syndrome. Science.

[187] Muzio L.L. (2008). Nevoid Basal Cell Carcinoma Syndrome (Gorlin Syndrome). Orphanet J. Rare Dis..

[188] Dahmane N., Lee J., Robins P., Heller P., Ruiz I Altaba A. (1997). Activation of the Transcription Factor Gli1 and the Sonic Hedgehog Signalling Pathway in Skin Tumours. Nature.

[189] Goodrich L.V., Milenković L., Higgins K.M., Scott M.P. (1997). Altered Neural Cell Fates and Medulloblastoma in Mouse Patched Mutants. Science.

[190] Vořechovský I., Tingby O., Hartman M., Strömberg B., Nister M., Collins V.P., Toftgård R. (1997). Somatic Mutations in the Human Homologue of Drosophila Patched in Primitive Neuroectodermal Tumours. Oncogene.

[191] Tostar U., Malm C.J., Meis-Kindblom J.M., Kindblom L.G., Toftgård R., Undén A.B. (2006). Deregulation of the Hedgehog Signalling Pathway: A Possible Role for the PTCH and SUFU Genes in Human Rhabdomyoma and Rhabdomyosarcoma Development. J. Pathol..

[192] Fan L., Pepicelli C.V., Dibble C.C., Catbagan W., Zarycki J.L., Laciak R., Gipp J., Shaw A., Lamm M.L.G., Munoz A. (2004). Hedgehog Signaling Promotes Prostate Xenograft Tumor Growth. Endocrinology.

[193] Tian H., Callahan C.A., Dupree K.J., Darbonne W.C., Ahn C.P., Scales S.J., De Sauvage F.J. (2009). Hedgehog Signaling Is Restricted to the Stromal Compartment during Pancreatic Carcinogenesis. Proc. Natl. Acad. Sci. USA.

[194] Ma X., Sheng T., Zhang Y., Zhang X., He J., Huang S., Chen K., Sultz J., Adegboyega P.A., Zhang H. (2006). Hedgehog Signaling Is Activated in Subsets of Esophageal Cancers. Int. J. Cancer.

[195] Zhao C., Chen A., Jamieson C.H., Fereshteh M., Abrahamsson A., Blum J., Kwon H.Y., Kim J., Chute J.P., Rizzieri D. (2009). Hedgehog Signalling Is Essential for Maintenance of Cancer Stem Cells in Myeloid Leukaemia. Nature.

[196] Turner K.A. (2017). Assessment of a Potential Therapeutic Target in the Hedgehog Pathway for the Eradication of Primitive Chronic Myeloid Leukemia Cells. Ph.D. Thesis.

[197] Wang X., Zhang N., Huo Q., Sun M., Dong L., Zhang Y., Xu G., Yang Q. (2014). Huaier Aqueous Extract Inhibits Stem-like Characteristics of MCF7 Breast Cancer Cells via Inactivation of Hedgehog Pathway. Tumor Biol..

[198] Varnat F., Duquet A., Malerba M., Zbinden M., Mas C., Gervaz P., Ruiz I Altaba A. (2009). Human Colon Cancer Epithelial Cells Harbour Active HEDGEHOG-GLI Signalling That Is Essential for Tumour Growth, Recurrence, Metastasis and Stem Cell Survival and Expansion. EMBO Mol. Med..

[199] Fu P., Zheng X., Fan X., Lin A. (2019). Role of Cytoplasmic LncRNAs in Regulating Cancer Signaling Pathways. J. Zhejiang Univ. Sci. B.

[200] Zhou H., Xiong Y., Peng L., Wang R., Zhang H., Fu Z. (2020). LncRNA-CCSC1 Modulates Cancer Stem Cell Properties in Colorectal Cancer via Activation of the Hedgehog Signaling Pathway. J. Cell. Biochem..

[201] Wu J., Zhu P., Lu T., Du Y., Wang Y., He L., Ye B., Liu B., Yang L., Wang J. (2019). The Long Non-Coding RNA LncHDAC2 Drives the Self-Renewal of Liver Cancer Stem Cells via Activation of Hedgehog Signaling. J. Hepatol..

[202] Guo L., Zhou Y., Chen Y., Sun H., Wang Y., Qu Y. (2018). LncRNA ASAP1-IT1 Positively Modulates the Development of Cholangiocarcinoma via Hedgehog Signaling Pathway. Biomed. Pharmacother..

[203] Peng W., Wu J., Fan H., Lu J., Feng J. (2017). LncRNA EGOT Promotes Tumorigenesis Via Hedgehog Pathway in Gastric Cancer. Pathol. Oncol. Res..

[204] Li L., Ma T.T., Ma Y.H., Jiang Y.F. (2019). LncRNA HCG18 Contributes to Nasopharyngeal Carcinoma Development by Modulating MiR-140/CCND1 and Hedgehog Signaling Pathway. Eur. Rev. Med. Pharmacol. Sci..

[205] Zhou M., Hou Y., Yang G., Zhang H., Tu G., Du Y.E., Wen S., Xu L., Tang X., Tang S. (2016). LncRNA-Hh Strengthen Cancer Stem Cells Generation in Twist-Positive Breast Cancer via Activation of Hedgehog Signaling Pathway. Stem Cells.

[206] He B., Peng F., Li W., Jiang Y. (2019). Interaction of LncRNA-MALAT1 and MiR-124 Regulates HBx-Induced Cancer Stem Cell Properties in HepG2 through PI3K/Akt Signaling. J. Cell. Biochem..

[207] Gao X.F., He H.Q., Zhu X.B., Xie S.L., Cao Y. (2019). LncRNA SNHG20 Promotes Tumorigenesis and Cancer Stemness in Glioblastoma via Activating PI3K/Akt/MTOR Signaling Pathway. Neoplasma.

[208] Ding K., Liao Y., Gong D., Zhao X., Ji W. (2018). Effect of Long Non-Coding RNA H19 on Oxidative Stress and Chemotherapy Resistance of CD133+ Cancer Stem Cells via the MAPK/ERK Signaling Pathway in Hepatocellular Carcinoma. Biochem. Biophys. Res. Commun..

[209] Cheng Z., Lei Z., Yang P., Si A., Xiang D., Zhou J., Hüser N. (2019). Long Non-Coding RNA THOR Promotes Liver Cancer Stem Cells Expansion via β-Catenin Pathway. Gene.

[210] Amelio I., Bernassola F., Candi E. (2021). Emerging Roles of Long Non-Coding RNAs in Breast Cancer Biology and Management. Semin. Cancer Biol..

[211] Cheng S., Wang L., Deng C.H., Du S.C., Han Z.G. (2017). ARID1A Represses Hepatocellular Carcinoma Cell Proliferation and Migration through LncRNA MVIH. Biochem. Biophys. Res. Commun..

[212] Zhao Y., Li Y., Sheng J., Wu F., Li K., Huang R., Wang X., Jiao T., Guan X., Lu Y. (2019). P53-R273H Mutation Enhances Colorectal Cancer Stemness through Regulating Specific LncRNAs. J. Exp. Clin. Cancer Res..

[213] Gao Y., Li X., Zhi H., Zhang Y., Wang P., Wang Y., Shang S., Fang Y., Shen W., Ning S. (2019). Comprehensive Characterization of Somatic Mutations Impacting LncRNA Expression for Pan-Cancer. Mol. Ther.-Nucleic Acids.

[214] Miao Y.R., Liu W., Zhang Q., Guo A.Y. (2018). LncRNASNP2: An Updated Database of Functional SNPs and Mutations in Human and Mouse LncRNAs. Nucleic Acids Res..

[215] Ning S., Zhang J., Wang P., Zhi H., Wang J., Liu Y., Gao Y., Guo M., Yue M., Wang L. (2016). Lnc2Cancer: A Manually Curated Database of Experimentally Supported LncRNAs Associated with Various Human Cancers. Nucleic Acids Res..

[216] Lee Y.W., Chen M., Chung I.F., Chang T.Y. (2021). LncExplore: A Database of Pan-Cancer Analysis and Systematic Functional Annotation for LncRNAs from RNA-Sequencing Data. Database.

[217] Zhou H., Feng B., Abudoureyimu M., Lai Y., Lin X., Tian C., Huang G., Chu X., Wang R. (2020). The Functional Role of Long Non-Coding RNAs and Their Underlying Mechanisms in Drug Resistance of Non-Small Cell Lung Cancer. Life Sci..

[218] Chen J., Lin C., Yong W., Ye Y., Huang Z. (2015). Calycosin and Genistein Induce Apoptosis by Inactivation of HOTAIR/p-Akt Signaling Pathway in Human Breast Cancer MCF-7 Cells. Cell. Physiol. Biochem..

[219] Guo W., Liu S., Cheng Y., Lu L., Shi J., Xu G., Li N., Cheng K., Wu M., Cheng S. (2016). ICAM-1-Related Noncoding RNA in Cancer Stem Cells Maintains ICAM-1 Expression in Hepatocellular Carcinoma. Clin. Cancer Res..

[220] Chen G., Cao Y., Zhang L., Ma H., Shen C., Zhao J. (2017). Analysis of Long Non-Coding RNA Expression Profiles Identifies Novel LncRNA Biomarkers in the Tumorigenesis and Malignant Progression of Gliomas. Oncotarget.

[221] Lin L.Y., Yang L., Zeng Q., Wang L., Chen M.L., Zhao Z.H., Ye G.D., Luo Q.C., Lv P.Y., Guo Q.W. (2018). Tumor-Originated Exosomal LncUEGC1 as a Circulating Biomarker for Early-Stage Gastric Cancer. Mol. Cancer.

[222] Wang Y., Lin X., Fu X., Yan W., Lin F., Kuang P., Luo Y., Lin E., Hong X., Wu G. (2018). Long Non-Coding RNA BANCR Regulates Cancer Stem Cell Markers in Papillary Thyroid Cancer via the RAF/MEK/ERK Signaling Pathway. Oncol. Rep..

[223] Wang Y., Zhu P., Luo J., Wang J., Liu Z., Wu W., Du Y., Ye B., Wang D., He L. (2019). LncRNA HAND2-AS1 Promotes Liver Cancer Stem Cell Self-Renewal via BMP Signaling. EMBO J..

[224] Ouyang S., Zhou X., Chen Z., Wang M., Zheng X., Xie M. (2019). LncRNA BCAR4, Targeting to MiR-665/STAT3 Signaling, Maintains Cancer Stem Cells Stemness and Promotes Tumorigenicity in Colorectal Cancer. Cancer Cell Int..

[225] Tang T., Guo C., Xia T., Zhang R., Zen K., Pan Y., Jin L. (2019). LncCCAT1 Promotes Breast Cancer Stem Cell Function through Activating WNT/β-Catenin Signaling. Theranostics.

[226] Mo M., Ma X., Luo Y., Tan C., Liu B., Tang P., Liao Q., Liu S., Yu H., Huang D. (2022). Liver-Specific LncRNA FAM99A May Be a Tumor Suppressor and Promising Prognostic Biomarker in Hepatocellular Carcinoma. BMC Cancer.

[227] Bhan A., Soleimani M., Mandal S.S. (2017). Long Noncoding RNA and Cancer: A New Paradigm. Cancer Res..

